# Small-Molecule Fluorescent Ligands for the CXCR4 Chemokine Receptor

**DOI:** 10.1021/acs.jmedchem.3c00151

**Published:** 2023-03-21

**Authors:** Sebastian Dekkers, Birgit Caspar, Joëlle Goulding, Nicholas D. Kindon, Laura E. Kilpatrick, Leigh A. Stoddart, Stephen J. Briddon, Barrie Kellam, Stephen J. Hill, Michael J. Stocks

**Affiliations:** † Biodiscovery Institute, School of Pharmacy, University of Nottingham, Nottingham NG7 2RD, U.K.; ‡ Centre of Membrane Proteins and Receptors, University of Birmingham and University of Nottingham, The Midlands NG7 2UH, U.K.; § Division of Physiology, Pharmacology & Neuroscience, Medical School, University of Nottingham, Nottingham NG7 2UH, U.K.

## Abstract

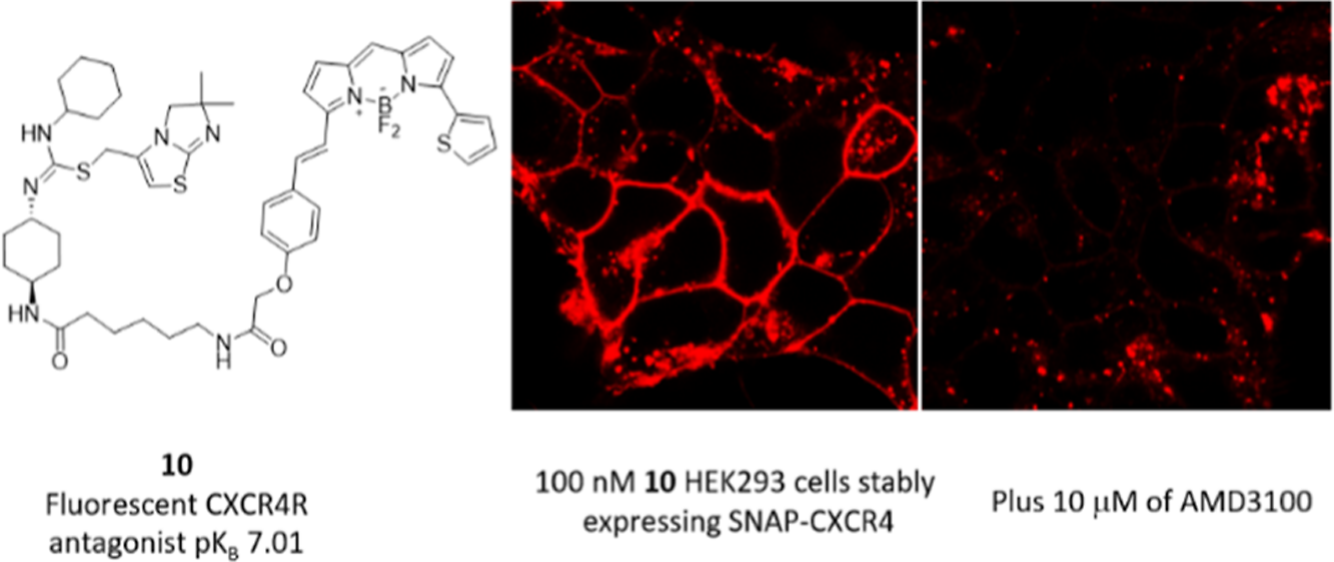

The C–X–C chemokine receptor type 4, or CXCR4, is a chemokine receptor found to promote cancer progression and metastasis of various cancer cell types. To investigate the pharmacology of this receptor, and to further elucidate its role in cancer, novel chemical tools are a necessity. In the present study, using classic medicinal chemistry approaches, small-molecule-based fluorescent probes were designed and synthesized based on previously reported small-molecule antagonists. Here, we report the development of three distinct chemical classes of fluorescent probes that show specific binding to the CXCR4 receptor in a novel fluorescence-based NanoBRET binding assay (p*K*_D_ ranging 6.6–7.1). Due to their retained affinity at CXCR4, we furthermore report their use in competition binding experiments and confocal microscopy to investigate the pharmacology and cellular distribution of this receptor.

## Introduction

The chemokine receptor CXCR4 is a membrane-bound protein belonging to the class A G protein-coupled receptors. It is a highly conserved protein, showing 99% sequence homology between human and murine species.^1^ CXCR4 is predominantly expressed on hematopoietic cells such as neutrophils, monocytes, and macrophages, but it is also found on neuronal stem cells, astrocytes, and micro-glia.^2^ Activation of CXCR4 by the chemokine CXCL12, its only endogenous ligand, results in a variety of physiological and cellular effects. Most importantly, activation of the CXCR4–CXCL12 axis induces the migration of CXCR4-expressing cells along CXCL12 gradients, homing immune cells to sites of inflammation. CXCR4 has also been found to play an essential role in embryonic development, as studies have shown that CXCR4-knockout mice display defects in B cell lymphopoiesis and bone marrow colonization, as well as late gestational lethality.^3−5^ In recent years, it has become increasingly clear that the activation of the CXCR4–CXCL12 signaling axis also contributes to the proliferation, migration, and invasion of various cancer cell types.^6−8^ Whereas expression of this receptor in healthy cells is limited to only a few cell types, more than 20 different cancer types were found to express this chemokine receptor.^9^ To illustrate, a study by Müller^10^ found that, while healthy breast tissue showed very low levels of CXCR4, its expression was heavily upregulated in primary breast carcinoma. Additionally, known secondary tumor sites for primary breast carcinoma such as lung tissue, lymph nodes, bone marrow, and liver all contain abundant levels of CXCL12.^11^ This suggests that the molecules and mechanisms that regulate leukocyte trafficking may be hijacked by CXCR4-expressing tumor cells. This hypothesis was further supported by Alsayed^12^ and Greim,^13^ whose investigations of multiple myeloma and leukemia, respectively, showed metastatic cells homed to the same bone marrow sites as where hematopoietic stem cells and hematopoietic progenitor cells normally reside. With the increase in importance of CXCR4 as a therapeutic target for cancer, as well as its relevance in HIV-1 entry and several autoimmune diseases, comes a need for chemical tools to study this receptor. To fill this need, and to further investigate the CXCR4–CXCL12 signaling axis, we sought to develop high affinity and fluorescent antagonists.

As the CXCR4 receptor is a known target for the treatment of various diseases and disorders, including HIV and WHIM syndrome, efforts to develop small-molecule inhibitors have yielded many different chemical classes of highly potent antagonists.^14−16^ They have furthermore led to the clinical approval of one drug—AMD3100, and a number of other small molecules which are currently undergoing clinical trials.^17,18^

**Figure 1:**
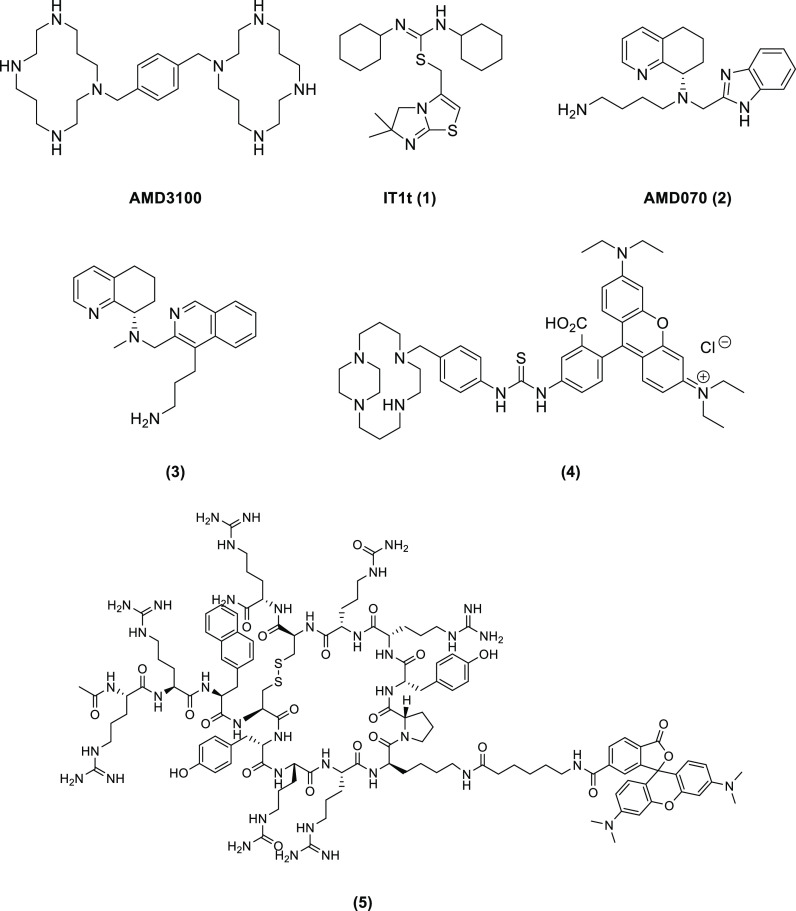
Selection of small-molecule CXCR4 receptor antagonists AMD3100 and **1–3** and fluorescent antagonists **4–5** relevant to this work.

Reported small-molecule inhibitors for CXCR4 (Figure 1) would make an excellent starting point for the development of novel fluorescent ligands. Some efforts were made to develop AMD3100-based fluorescent probes such as compounds **4**(19,20) and also peptide-based **5** (AcTZ14011TAMRA).^21^ However, due to a combination of either poor pharmacological properties or complex synthesis, their use is severely limited. The design of small-molecule-based fluorescent probes requires a rational approach, and several factors need to be taken into account.^22,23^ First, the point of attachment of the linker to the pharmacophore must be relatively insensitive to structural modification and must tolerate bulky substituents. Second, the type of linker, as well as the length that separates the orthostere from the fluorophore can affect the pharmacology of the resulting conjugate. Finally, the physiochemical properties of the final conjugate is assessed to minimize non-specific binding and to prevent high intracellular ligand accumulation.

In this study, our goal was to use a classic medicinal chemistry approach for the development of novel fluorescent probes based on the known CXCR4 receptor antagonists **1–3**. A thorough evaluation of the structure–activity relationship (SAR) of these selected small-molecule inhibitors, in combination with in silico design, informed the synthetic strategy for linker and fluorophore attachment. The resulting fluorescent conjugates would be characterized using a BRET-based assay, facilitated by the use of a NanoLuciferase (NLuc)-CXCR4 receptor construct. This NanoBRET methodology has recently been developed and has allowed characterization of various (fluorescent-) probes targeting GPCRs.^24,25^

## Results and Discussion

### IT1t-Based Fluorescent Probes

**Figure 2:**
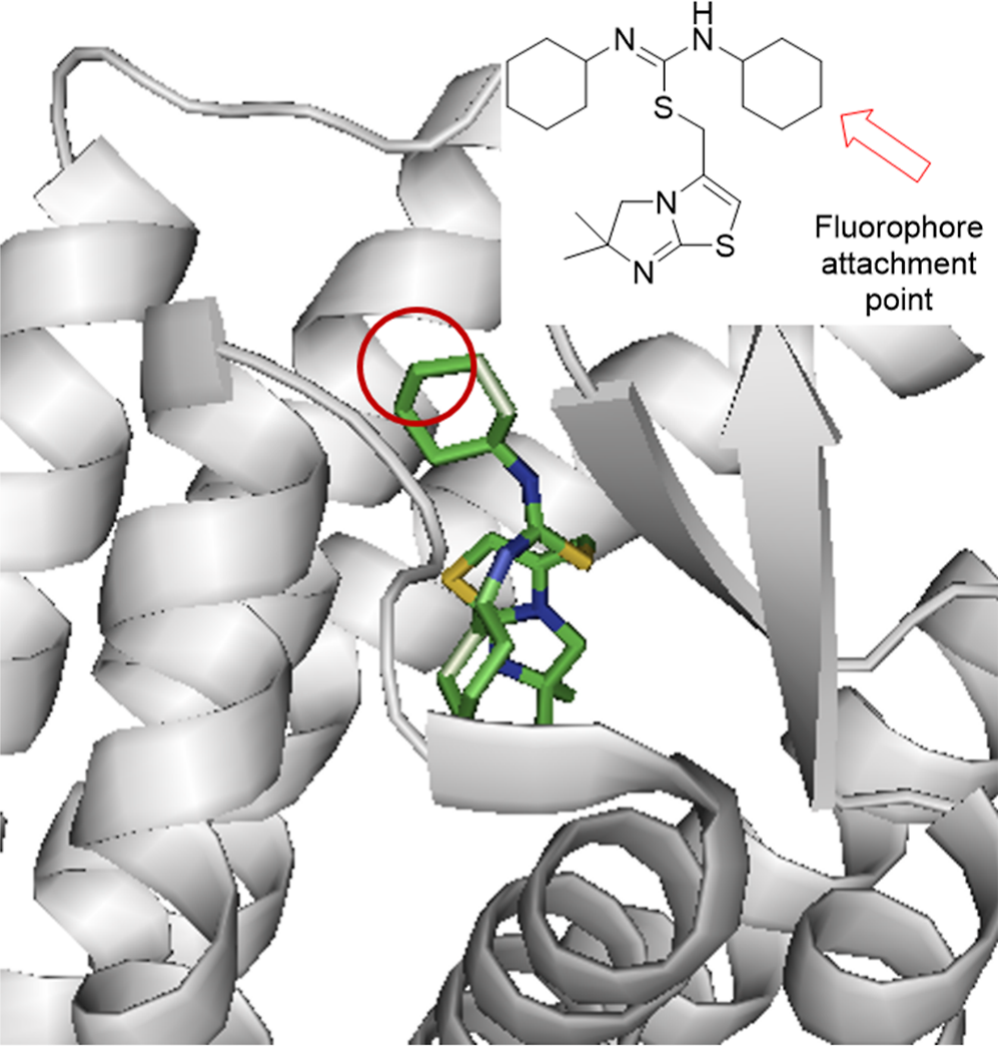
Crystal structure showing the CXCR4 receptor bound to IT1t (PDB ID: 3ODU).^26^ The arrow demonstrates the cyclohexyl group pointing out of the transmembrane domain modified to enable attachment of a fluorescent probe.

In 2010, Wu^26^ successfully resolved the crystal structure of CXCR4. In one of the two crystal structures, CXCR4 was complexed with the small molecule IT1t (Figure 2).

**Scheme 1:**
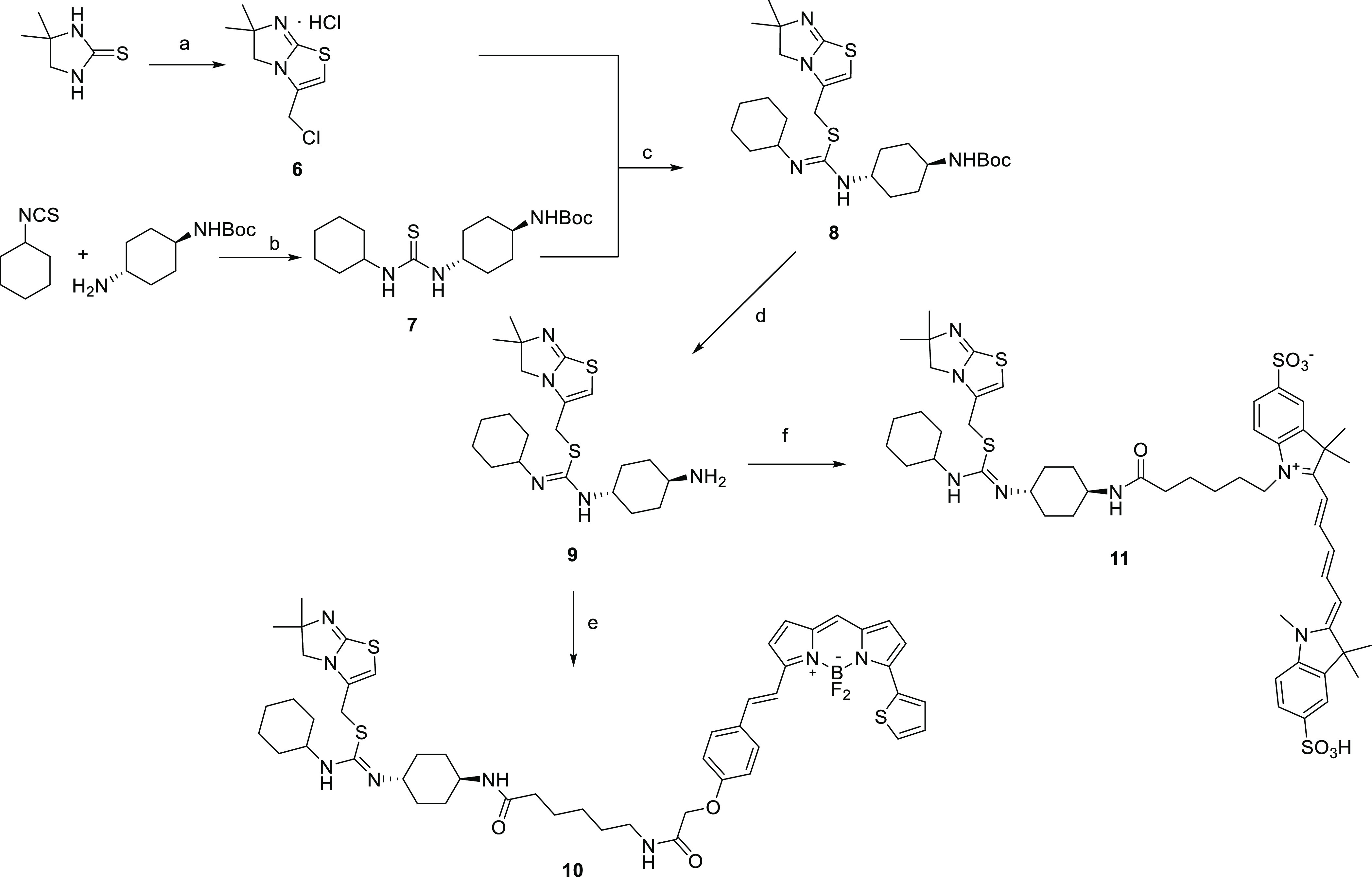
Synthesis of Amine-Substituted IT1t Derivatives Reagents and conditions: (a) two steps: (1) 1,3-dichloroacetone, acetonitrile, reflux, 2 h.; (2) diglyme, 140 °C, 2 h, 67% over two steps; (b) dichloromethane, 20 h rt, 85–98%; (c) acetonitrile, 16 h, reflux, 40–48%; (d) TFA, dichloromethane, 3 h, rt, quant.; (e) BODIPY-SE, DIPEA, acetonitrile, rt, 3–4 h, 62%; and (f) 1-(6-((2,5-dioxopyrrolidin-1-yl)oxy)-6-oxohexyl)-3,3-dimethyl-2-((1*E*,3*E*)-5-((*Z*)-1,3,3-trimethyl-5-sulfoindolin-2-ylidene)penta-1,3-dien-1-yl)-3*H*-indol-1-ium-5-sulfonate (sulfo-cyanine5 NHS ester), DIPEA, DMF, 16 h, 81%.

IT1t is part of a class of isothiourea CXCR4 receptor antagonists originally described by Thoma^27^ that were found to be highly potent (for IT1t, IC_50_ = 8.0 nM) displaying excellent bioavailability and in vivo activity. IT1t binds to a relatively shallow part of the orthosteric binding pocket, as compared to the endogenous ligand CXCL12, potentially allowing structural modification to incorporate a fluorophore. Furthermore, while the heteroatoms of the two isothiourea moieties of IT1t form salt bridges with residues Asp97 and Glu288 of CXCR4, the cyclohexane rings engage with the receptor through hydrophobic interactions, with one pointing out of the GPCR channel (Figure 2). Therefore, functionalization of one of these cyclohexane rings could allow tethering to a fluorescent dye, without compromising affinity. Precedent for the structural modification of one of these rings was illustrated by the development of glycomimetic structures as novel CXCR4 receptor inhibitors. Here, IT1t was used as a recognition element and was conjugated to glycomimetics using para-amino and *para*-carboxy groups.^28^ We thus envisioned that tethering off one of the cyclohexane rings would minimize the loss of receptor affinity, and doing so by introducing an amine outside the ring would allow for convenient conjugation to amino-reactive dyes such as succinimidyl ester-protected BODIPYs and sulfo-cyanine5 (see Scheme 1).

The synthesis of IT1t-based fluorescent probes started from commercially available 4,4-dimethyl-2-imidazolidinethione. A two-step reaction with dichloroacetone allowed for the conversion into isothiourea **6**.^27^ Separately, reaction of cyclohexyl isothiocyanate with *tert*-butyl (4-aminocyclohexyl)carbamate afforded intermediates **7** in excellent yield, and provided the necessary conjugation sites to a linker/fluorophore combination. Subsequent alkylation between key intermediates **6** and **7** under reflux conditions afforded the desired compound **8**, which after treating with trifluoracetic acid gave **9** ready for conjugation to a linker/fluorophore. Here, we opted to use the succinimidyl ester-protected BODIPY 630/650-X, which already incorporates a seven-atom aminohexanoyl spacer, allowing for the introduction of a linker and fluorophore in a single step to generate fluorescent IT1t derivative **10** and 1-(6-((2,5-dioxopyrrolidin-1-yl)oxy)-6-oxohexyl)-3,3-dimethyl-2-((1*E*,3*E*)-5-((*Z*)-1,3,3-trimethyl-5-sulfoindolin-2-ylidene)penta-1,3-dien-1-yl)-3*H*-indol-1-ium-5-sulfonate to give **11** in high yields after purification by reverse-phase HPLC (Scheme 1).

### Tetrahydroquinoline-Based Fluorescent Probes

When evaluating the SAR of other reported classes of CXCR4 receptor inhibitors, such as compounds **2** and **3**, it became evident that most compounds bear little resemblance to IT1t. Unlike the above mentioned, most CXCR4 inhibitors incorporate aromatic heterocycles and contain basic nitrogen atoms that form a so-called “nitrogen triad”.^15^ Recent docking studies performed using AMD070 (**2**), perhaps the best example of a stereotypical CXCR4 receptor antagonist, revealed that this triad forms crucial interactions with residues Glu288 and Asp187 within the crystal structure, and hydrophobic interactions are formed between the aromatic heterocycles of AMD070 and residues His294, Ile265, and Ser295.^29^ The primary amine was shown in one pose to interact with Asp97, which is part of transmembrane helix II and is located relatively close to the extracellular surface and, similarly to the piperidine-substituted IT1t derivative, causes the distal amine of the ligand to angle toward the solvate. Conjugation to a selected fluorophore directly attached to this amine, via the formation of an amide bond might potentially attenuate its ability to interact with Asp97.

Further exploration of the published SAR around AMD070 shows a shift of the distal amine from the central nitrogen atom to the benzimidazole ring.^30,31^ Furthermore, the use of an isoquinoline core instead of benzimidazole increased potency and improved ADME properties.^32^ Importantly, changing the length of the side-chain and functionalizing the primary amine did not markedly affect binding properties.^32^

**Figure 3:**
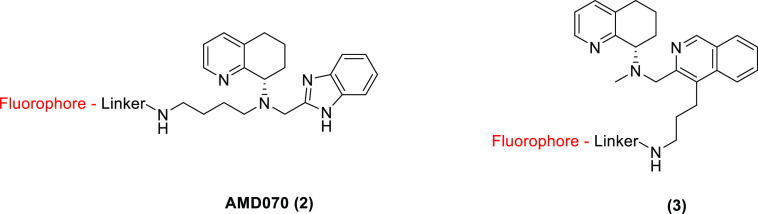
Fluorescent ligand design showing linker addition points for fluorescent conjugates.

These findings suggest that this flexible part of the molecule is highly tolerant toward substitution and is available to allow conjugation to a linker/fluorophore combination. Cognizant to the above, we set out to develop fluorescent derivatives of both the AMD070 **2** and the isoquinoline **3** (Figure 3).

**Scheme 2:**
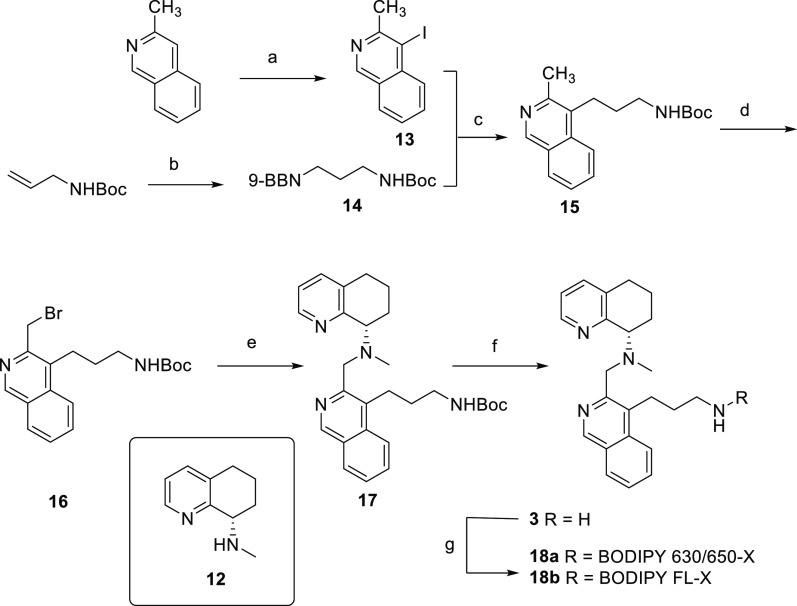
Synthesis of Fluorescent Derivatives of the Isoquinoline Scaffold Reagents and conditions: (a) glacial AcOH, NIS, 80 °C, 16 h, 52%; (b) 9-BBN 0.5 M in tetrahydrofuran, N_2_, 0 °C to rt, 3 h.; (c) Pd(PPh_3_)_4_, potassium hydroxide 1.0 M in H_2_O, tetrahydrofuran, 80 °C, 1 h, 27% over two steps; (d) NBS, AIBN, dichloroethane, N_2_, 80 °C, 4 h.; (e) **10**, DIPEA, acetonitrile, rt, 16 h, 21% over 2 steps; (f) TFA, dichloromethane, rt, 1 h, 49%; and (g) appropriate BODIPY-X NHS ester, DIPEA, acetonitrile, rt, 16 h, 51–55%.

As for both scaffold compound classes, it was reported that the (*S*)-isomer of the tetra-hydroquinoline ring displayed the higher affinity and potency toward the CXCR4 receptor,^30−32^ we felt it was necessary to develop enantiomerically pure fluorescent conjugates. The synthesis of these tetrahydroquinoline-based fluorescent probes therefore commenced with the synthesis of the chiral, amine-substituted tetrahydroquinoline moiety **12**.^31,33^ Following the procedures reported by Miller,^32^ electrophilic iodination of 3-methylisoquinoline with *N*-iodosuccinimide (NIS) afforded selectively the ortho-substituted product (**13**, Scheme 2). In a parallel fashion, hydroboration^34^ of *N*-allylcarbamate with 9-BBN allowed for subsequent Suzuki–Miyaura coupling to install the distal amine on the isoquinoline core. Bromomethyl intermediate **16** prepared by radical bromination was immediately reacted with the chiral fragment **12**. Finally, after *N*-Boc removal by TFA, the congener was conjugated to both a red fluorescing BODIPY 630/650-X (**18a**) and a green fluorescing BODIPY FL-X (**18b**). Introduction of the smaller, green fluorescing BODIPY allows us to investigate the effect of the fluorophore size on ligand binding, as well as expanding the available toolkit for studying the CXCR4 receptor.

**Scheme 3:**
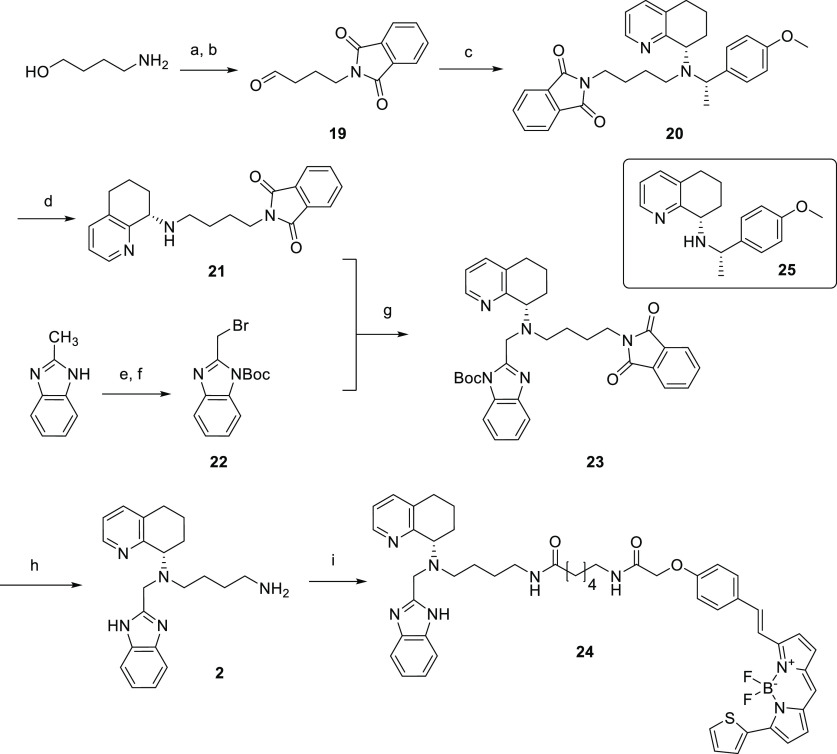
Synthesis of a Fluorescent AMD070 Derivative Reagents and conditions: (a) phthalic anhydride, toluene, 4 Å mol. sieves, reflux, 3 h, 53%; (ii) Dess–Martin periodinane, dichloromethane, 0 °C—rt, 2 h 74%; (c) **23**, NaBH(OAc)_3_, dichloromethane, N_2_, rt, 16 h, 69%; (d) TFA, dichloromethane, rt, 1 h, quant.; (e) Boc_2_O, TEA, DMAP, dichloromethane, rt, 5 min, 95%; (f) NBS, AIBN, dichloroethane, 95 °C, 6 h, 66%; (g) TEA, KI, acetonitrile, MW, 80 °C, 1 h, 27%; (h) hydrazine, ethanol, MW, 80 °C, 1 h, 84%; and (i) BODIPY-X 630/650-NHS ester, DIPEA, acetonitrile, rt, 16 h, 53%.

Synthesis of a fluorescent AMD070 derivative (Scheme 3) started from *N*-phthalimide protection of 4-amino-1-butanol and subsequent oxidation of the alcohol to the aldehyde **19**. Reductive amination of **19** with intermediate **25**(33) introduced the chiral tetrahydroquinoline fragment to the distal amine. Separately, *N*-Boc protection of 2-methylbenzimidazole and subsequent radical bromination afforded intermediate **22** in good yield. *N*-alkylation of this alkyl-bromide with key intermediate **20** finally assembled the scaffold, which, after simultaneous removal of the phthalimide and *N*-boc with hydrazine, was coupled to the red fluorescing BODIPY 630/650-X to give fluorescent ligand **24**.

All fluorescent ligands synthesized from these three scaffolds were isolated and purified by preparative RP-HPLC, and their purities confirmed to be >95% by analytical RP-HPLC. Furthermore, the chemical identity of these probes (**10**, **11**, **18a–b**, and **24**) was confirmed using high-resolution mass spectrometry (HRMS TOF ES^+^).

### Pharmacological Evaluation of the Fluorescent CXCR4 Antagonists

**Figure 4:**
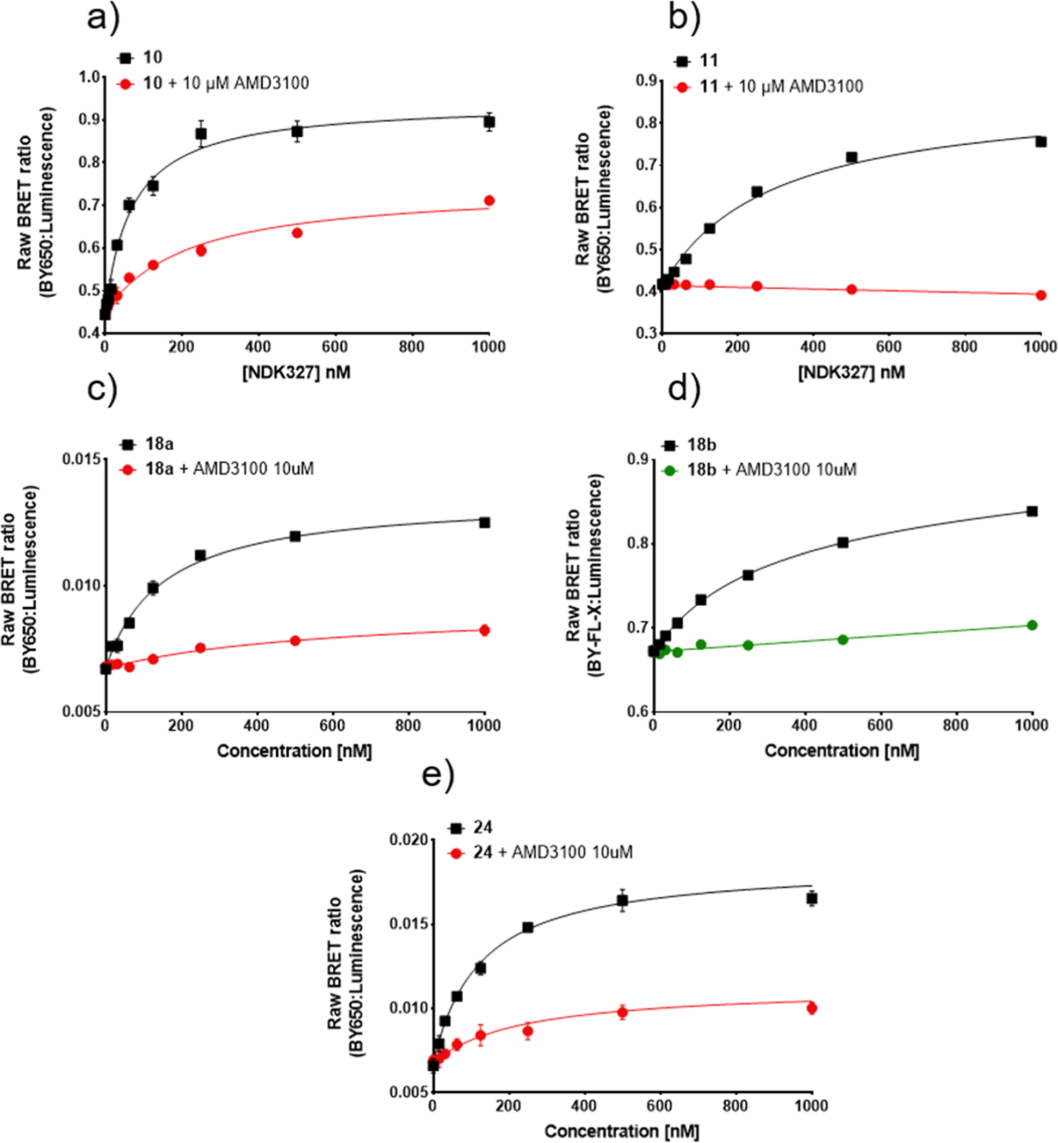
NanoBRET saturation binding curves measured in HEK293G NLuc-CXCR4 cell membranes for (a) **10**, (b) **11**, (c) **18a**, (d) **18b**, and (e) **24**. Raw BRET ratio was determined by the ratio of fluorescence/luminescence emissions. Measurements were made in the absence (total binding) and presence of 10 μM AMD3100. For those curves (a,c,e) where the non-specific binding curves obtained in the presence of 10 μM AMD3100 were not linear, pKd values were estimated by fitting the total binding curves to a saturable plus linear component. Data points represent mean ± S.D. of triplicate determinations of a single experiment. These single experiments are representative of 3–5 separate experiments per fluorescent ligand. Where not seen, error bars are within the symbol. White opaque-bottom 96-well plates were used for compounds **10** and **11** (a,b), whereas white clear-bottom 96-well plates were used for compounds **18a**, **18b**, and **24** (c–e).

The above synthesized fluorescent conjugates were assessed in a variety of pharmacological assays. First, to determine whether the fluorescent conjugates maintained their affinity toward the CXCR4 receptor, saturation binding experiments were performed. Here, the fluorescent properties of these compounds allowed us to report the proximity of the fluorescent ligands to a N terminal NanoLuciferase-tagged receptor (NLuc-CXCR4) using bioluminescence resonance energy transfer (BRET).^24^ The four fluorescent conjugates produced clear saturable specific binding to the CXCR4 receptor that was associated with low levels of non-specific binding, resulting in p*K*_D_ values ranging from 7.07 to 6.45 (Table 1 and Figure 4).

**Table 1:** Binding Affinities of the Novel Fluorescent Ligands Determined in Membranes from HEK293G Cells Stably Expressing NLuc-CXCR4

example	p*K*_D_ (log *M*)^a^	*n*
**10** (BY630/650-X)	7.01 ± 0.11	4
**11** (sulfo-cyanine5)	6.46 ± 0.05	5
**18a** (BY630/650-X)	6.82 ± 0.09	3
**18b** (BY-FL-X)	6.62 ± 0.08	4
**24** (BY630/650-X)	7.07 ± 0.03	4

a p*K*_D_ value was calculated from the negative logarithm of the equilibrium dissociation constant (*K*_D_) determined from saturation-binding experiments using increasing concentrations of labeled ligand in the absence or presence of AMD3100 (10 μM). Data are expressed as mean ± SEM of n experiments, where each experiment was performed in triplicate.

Pleasingly, IT1t-based fluorescent compound **10** showed clear concentration-dependent increases in BRET (Figure 4a), reaching saturation at about 0.5 μM. In addition, the sulfo CY5 fluorescent analogue compound **11**, also showed a very clear concentration-dependent increases in BRET (Figure 4b) reaching saturation at about 1.0 μM with less visible non-specific binding compared to compound **10**, resulting from non-specific BODIPY accumulation due to the lower lipophilicy and higher solubility of the sulfo CY5 fluorophore. It was notable that the curve for compound **10** obtained in the presence of 10 μM AMD3100 was not linear (and still retained a detectable saturable component of binding). This suggests that there was incomplete displacement of specific binding by AMD3100 at the concentration used which might be suggestive of a non-competitive interaction (see below).

Compounds **18a** and **18b**, based on the isoquinoline scaffold, were both able to produce a concentration-dependent increase in BRET ratio, with compound **18a** reaching saturation at about 0.5 μM, and compound **18b** reaching saturation above 1.0 μM (Figure 4c,d, respectively). Interestingly, compound **18b** produced a substantially larger BRET ratio than compound 1**8a**. Though this may be attributed to a variety of factors, it is mainly thought to be due to the choice of acceptor fluorophore. When using green fluorescing ligands (503/512 nm excitation and emission, respectively, for BODIPY-FL-X), there is a greater spectral overlap between NLuc emission (emission peak 462 nm) and the fluorophore.^24^ This spectral overlap results in emitted light of the NLuc also being recorded in the acceptor emission channel, adding background noise. For the red fluorescent BODIPY 630/650-X dye, there is greater separation between its excitation and emission wavelengths and the NLuc emission wavelength, contributing to lower background noise.^24^

Lastly, the fluorescent AMD070 derivative **24** (Figure 4e) also displayed a clear saturable concentration-dependent BRET signal with a p*K*_D_ value higher than that of **18a**. Interestingly, both ligands contain the same fluorophore and pharmacophores, which are chemotypically similar. It is possible that **24** binds the CXCR4 receptor more easily due to the more preferred position of the distal amine, as well as its more flexible nature. It was also notable that, similar to compound **10** above, the binding of increasing concentration of **24** obtained in the presence of 10 μM AMD3100 was not linear (Figure 4e).

**Figure 5:**
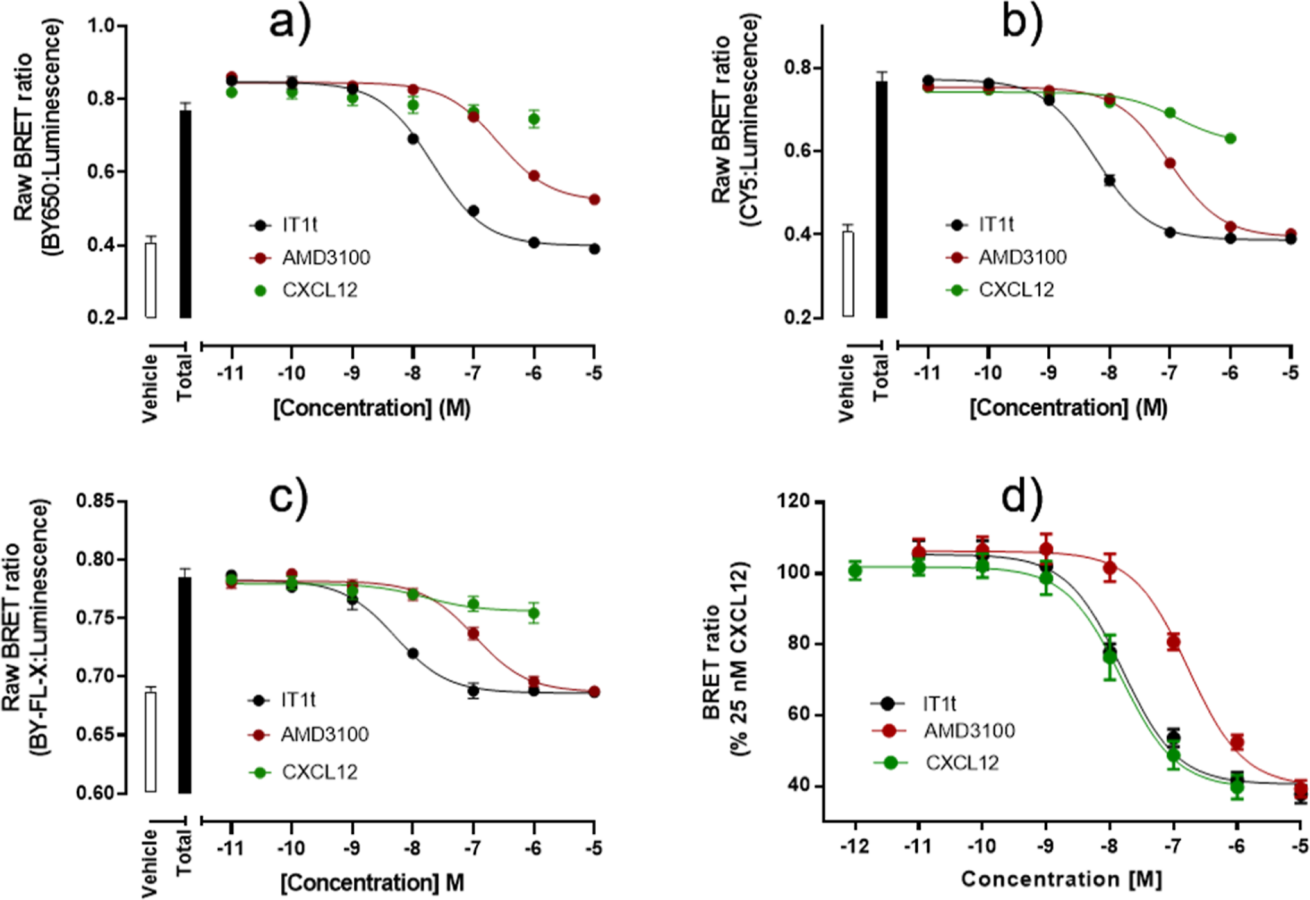
NanoBRET signal from NLuc-CXCR4 cell membranes treated with (a) 100 nM compound **10**, (b) 350 nM compound **11**, (c) 350 nM compound **18b**, (d) 25 nM CXCL12AF-647, and increasing concentrations of CXCL12, AMD3100, and IT1t. Raw BRET ratio was determined as the ratio of fluorescence/luminescence emissions. Data shown represent mean ± S.D. of *n* = 5 (a), *n* = 5 (b), *n* = 4–3 (c), and *n* = 5 (d), where each separate experiment was performed in triplicate.

Expanding the CXCR4 receptor toolkit was one of the primary objectives of our study. It is therefore imperative to evaluate the fluorescent conjugates developed in this study for their ability to be used as “hot” ligands in screening assays for novel (small molecule) effectors. Compounds **10**, and **11** and **18b** were therefore selected to be used in NanoBRET competition binding experiments, alongside Alexa Fluor 647-labeled CXCL12 (CXCL12AF-647) whose use as a fluorescent probe has already been shown^35,36^ (Figure 5).

HEK293G cell membranes expressing NLuc-CXCR4 were therefore incubated with **10** (100 nM, Figure 5a), **11** (350 nM, Figure 5b), **18b** (250 nM, Figure 5c) and CXCL12AF-647 (25 nM, Figure 5d), and increasing concentrations of known small molecules IT1t and AMD3100, as well as the endogenous ligand CXCL12. Using the small-molecule-based fluorescent probes, a concentration-dependent decrease of the BRET signal was observed for the both IT1t and AMD3100, but not for CXCL12—suggesting the fluorescent conjugates bind in a non-competitive manner with regards to CXCL12. Fluorescently labeled CXCL12 was fully displaced by unlabeled CXCL12, showing the effect observed in Figure 5a–c is indeed ligand-specific. As the unlabeled small molecules IT1t and AMD3100 displayed a clear concentration-dependent decrease of the BRET signal, binding affinities could be estimated (Table 2). In the case of the interaction between AMD3100 and the IT1t derivative **10**, a full displacement of specific binding was not achieved even at the highest concentrations of AMD3100 used. This is in keeping with a non-competitive interaction suggested by the saturation obtained in Figure 4a.

**Table 2:** Binding Affinities (p*K*_i_) of CXCL12, AMD3100, and IT1t as Calculated from Cheng–Prusoff Analysis of the Data Displayed in Figure 4

	IT1t		AMD3100			CXCL12	
example	pIC_50_	p*K*_i_	pIC_50_	p*K*_i_	% max. inhibition of AMD3100^a^	pIC_50_	p*K*_i_
**10**	7.70 ± 0.13 (4)	8.04 ± 0.11 (4)	6.47 ± 0.20 (4)	N.D	75.26 ± 7.08	N.D	N.D
**11**	8.18 ± 0.12 (4)	8.50 ± 0.07 (4)	7.16 ± 0.11 (4)	7.45 ± 0.07 (4)	95.20 ± 9.60	N.D	N.D
**18b**	7.57 ± 0.36 (3)	8.59 ± 0.06 (3)	6.82 ± 0.66 (3)	7.29 ± 0.06 (3)	96.25 ± 3.82	N.D	N.D
CXCL12^AF-647^		8.04 ± 0.01 (5)		7.00 ± 0.04 (5)			7.97 ± 0.07 (5)

a Maximum inhibition of AMD3100 in respect to IT1t, calculated following baseline removal of BRET ratios measured at 10–11 M. Data were then normalized as a percentage of maximum inhibition seen at 10–5 M IT1t (100%). All values are expressed as mean ± S.E.M. Number of experiments are shown in parenthesis.

Overall, these NanoBRET competition-binding experiments show that small-molecule based fluorescent conjugates make excellent tool compounds for the characterization of small-molecule CXCR4 receptor antagonists. Conjugate **11**, in particular displayed excellent characteristics, as little background BRET was observed, and the pKi values obtained for IT1t and AMD3100 highly resemble values obtained for CXCL12AF-647.

**Figure 6:**
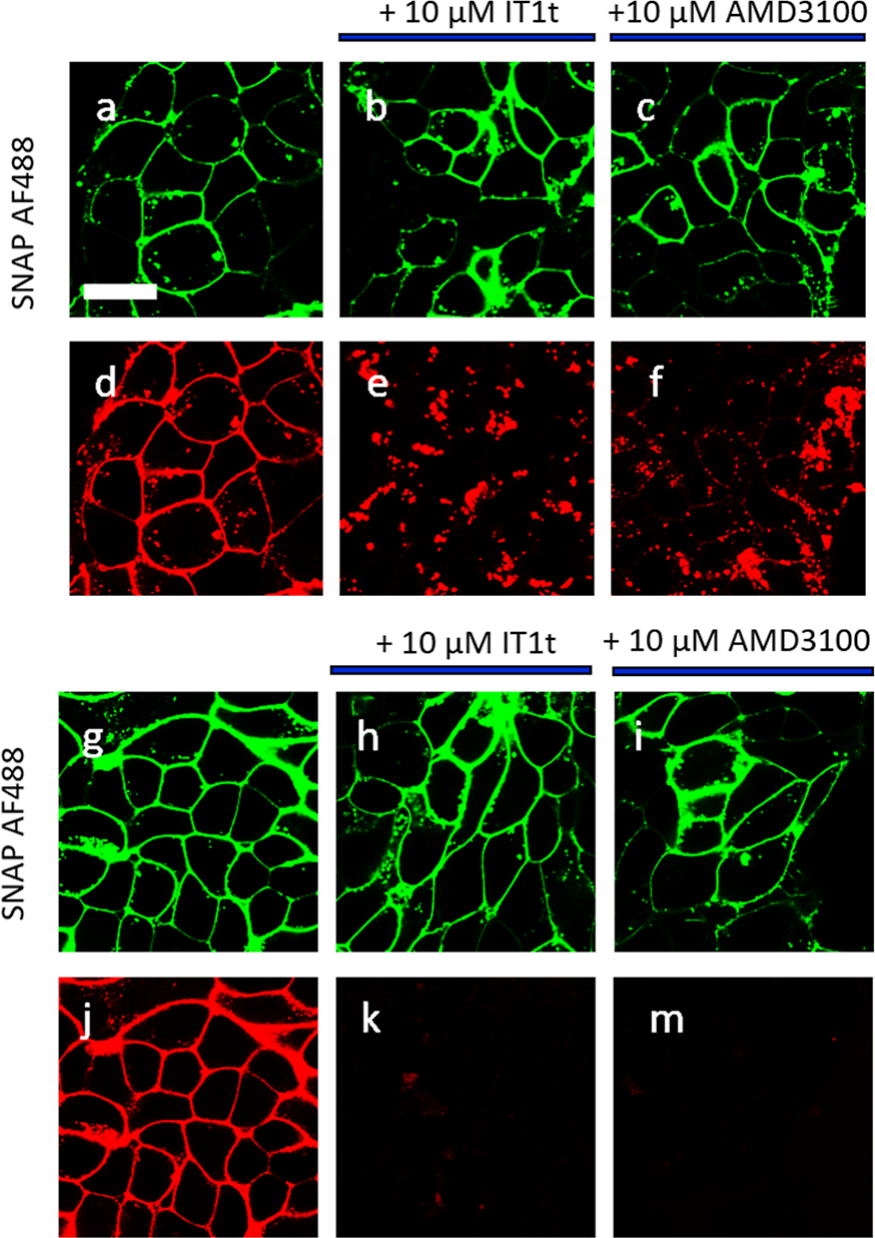
Visualization of the binding of 100 nM **10** (d–f) and **11** (j–m) to HEK293G cells stably expressing SNAP-CXCR4, scale bar = 20 μm. (a–c,g–i) Snap-Surface Alexa Fluor 488 labelling of SNAP-CXCR4. Fluorescent ligand binding in the (d,j) absence or (e,k) presence of 10 μM It1t or (f,m) 10 μM AMD3100 (30 min pre-incubation). Single equatorial confocal images were obtained after 30 min incubation at 37 °C in the continued presence of the fluorescent ligand (**10–11**) and unlabeled antagonist if present. Images shown are from a single experiment representative of at least three performed.

The availability of red fluorescent CXCR4 receptor ligands with reasonable affinities suggested that they may have utility for imaging of the receptor in living cells. Confocal microscopy images of fluorescent ligands **10**, **11**, **18a**, and **24** with HEK293G cells stably expressing N terminal SNAPTag-CXCR4 (referred to as SNAP-CXCR4) were captured. Ligand **10** showed localized membrane fluorescence and very little intracellular fluorescence at 100 nM ligand concentration (Figure 6). When cells were pretreated with either IT1t or AMD3100, the specific-membrane fluorescence of **10** was substantially reduced, indicating that the majority of the membrane fluorescence observed was specific labeling of the CXCR4 receptor. In the case of ligand 11, the fluorescence was highly localized to the cell membrane and this was completely prevented by IT1t and AMD3100 (Figure 6).

**Figure 7:**
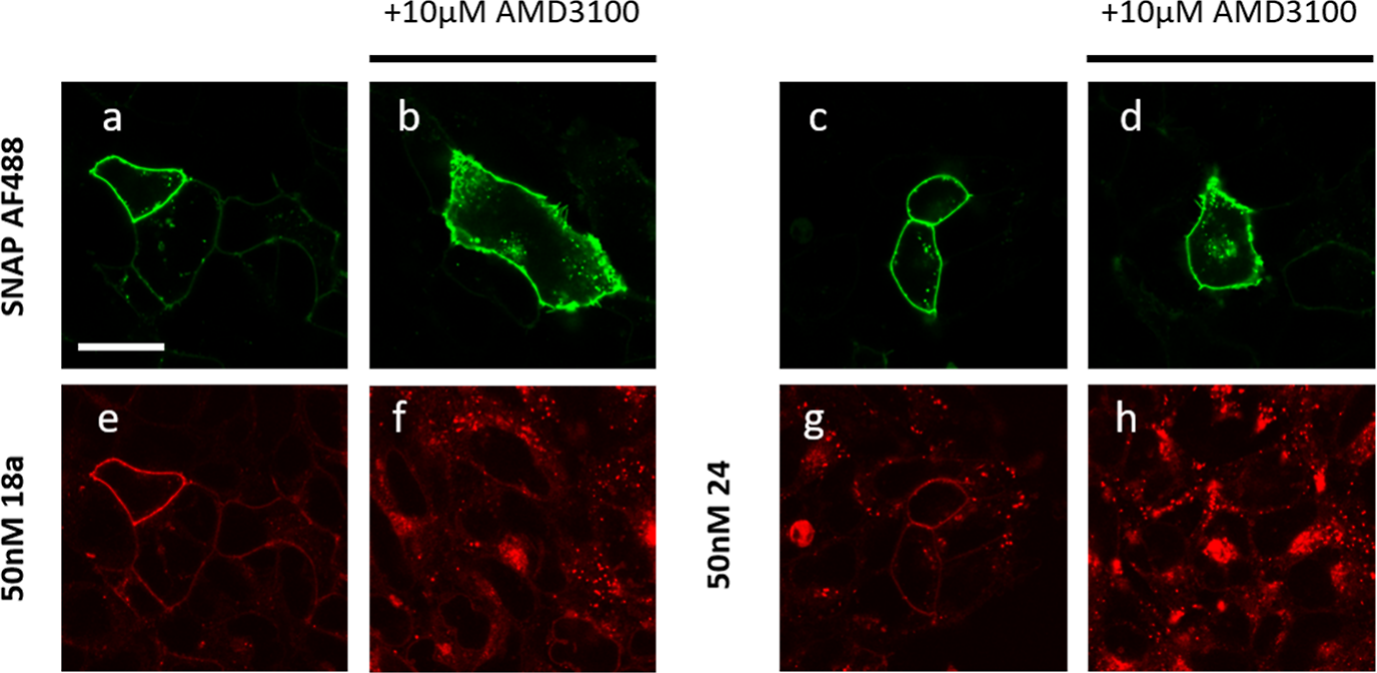
Visualization of the binding of **18a** and **24** at 50 nM on HEK293G cells stably expressing SNAP-CXCR4, scale bar = 20 μm. (a–d) Snap-Surface Alexa Fluor 488 labelling of SNAP-CXCR4. (e,f) 50 nM **18a** and (g,h) 50 nM **24** binding in the (e,g) absence or (f,h) presence of 10 μM AMD3100 (30 min pre-incubation). Single equatorial confocal images were obtained after 30 min incubation at 37 °C in the continued presence of the fluorescent ligand and unlabeled antagonist if present. Images shown are from a single experiment representative of at least three performed.

Ligands **18a** and **24** likewise showed reasonable membrane fluorescence; however, intracellular fluorescence was also apparent at 50 nM, demonstrating a higher degree of membrane penetration by the lipophilic ligand and increased non-specific binding. Treatment with AMD3100 reduced binding to cell surface receptors but this was accompanied by an increase in the level of intracellular fluorescence and punctate labeling on the cell surface of cells (Figure 7).

The confocal images clearly demonstrate that the less lipophilic sulfo CY5 fluorescent ligand based on the IT1t scaffold **11** possesses less non-specific membrane binding than either **10**, which in turn has less non-specific membrane binding than **18a** or **24**. This may be due in part to the physicochemical properties of the ligands as it is well known that dibasic compounds, such as **18a** and **24**, have a propensity to distribute into cellular membranes.^37−39^

## Conclusions

In the present study, we have reported the successful development and characterization of new small-molecule-based fluorescent probes for the CXCR4 receptor. A series of diverse small-molecule antagonists were selected and based on a combination of SAR analysis and in silico docking were synthesized to incorporate fluorescent BODIPY and sulfo CY5 dyes. The fluorescent conjugates retained good affinity toward the CXCR4 receptor, as determined by NanoBRET saturation experiments. We furthermore showed that the IT1t-based fluorescent conjugates **10** or **11** make excellent candidates as screening tools for new CXCR4 antagonists, with **10** being preferred, in part due to its higher affinity. In NanoBRET competition-binding experiments **10** and **11** displayed good signal-to-noise being displaced by established small-molecule antagonists IT1t and AMD3100. It should be noted that the sulfo CY5 fluorescent compound **11**, demonstrated less non-specific membrane binding presumably due to its lower lipophilicity and should be considered for confocal imaging. This observation was also supported from the BRET saturation data between compounds **10** and **11** (Figure 4a,b).

The novel CXCR4 receptor ligands developed here can be used in a range of fluorescence-based techniques. Through the incorporation of BODIPY and sulfo CY5 dyes, these ligands can be used in whole cell confocal microscopy experiments and in combination with the NanoBRET approach may help to shed light on the functionality of CXCR4 and its involvement in pathophysiological conditions.

## Experimental Section

### Chemistry: Materials and Methods

HPLC grade and analytical grade chemicals and solvents were purchased from the standard suppliers and were used without further purification. BODIPY and sulfo CY5 fluorophores were obtained from Molecular Probes (Fischer Scientific, U.K.). Sigma-Aldrich supplied high-grade silica, 60 Å, 230–400 mesh, for flash chromatography, and deuterated solvents (chloroform-*d*, methanol-*d*_4_, and DMSO-*d*_6_) were purchased from Sigma-Aldrich. Reactions were monitored by thin-layer chromatography (TLC) on commercially available silica precoated aluminum-backed plates (Merck Kieselgel 60 F^254^). Visualization was under UV light (254 and 366 nm), and where necessary staining with ninhydrin or potassium permanganate dips. For reactions requiring elevated pressure and temperatures, 10 or 35 mL reaction vessels were loaded into a Discover SP microwave (CEM, USA). The required duration and temperatures of the reactions were programmed using the software Synergy, which calculated the amount of power that was needed to perform the reaction. NMR spectra were recorded with a Bruker AV(III) 400 NMR spectrometer. ^1^H NMR was recorded at 400.13 MHz, and ^13^C NMR was recorded at 101.6 MHz. Deuterated solvents used for the preparation of NMR samples were CDCl_3_, MeOD-*d*_4_, or DMSO-*d*_6_. Chemical shifts (δ) are reported in ppm with reference to the chemical shift of the deuterated solvent. Coupling constants (*J*) are recorded in hertz, and the signal multiplicities are described by the following: s, singlet; d, doublet; t, triplet; q, quartet; brs, broad singlet; m, multiplet; dd, doublet of doublets; ddd, double doublet of doublets; dt, doublet of triplets; and p, pentet. NMR data were processed using MestReNova version 10.0.2. For the analysis of reaction mixtures and isolated compounds, a Shimadzu UFLCXR HPLC was used, equipped with a Biosystems MDS SCIEX API2000 ESI+ MS. The column was a Gemini 3 μm C18 110 Å, LC column 50 × 2 nm. As eluent, a mixture of MeCN and H_2_O was used, containing 0.1% formic acid. Samples were run using a gradient of 1:19 v/v to 19:1 v/v over either 5 or 15 min, with a flowrate of 0.5 mL/min. UV absorption was detected at 254 and 220 nm. Preparative RP-HPLC was performed on a Waters 2767 sample manager coupled to Waters 2525 binary-gradient module and a Waters 2457 dual-wavelength absorbance detector. The column used was a Phenomenex Gemini-NX (5 μm, 110 Å, C18, 150 × 21 mm) at ambient temperature. The flow rate was 25 mL/min, and UV detection was at 254 nm. Mobile phases were solvent A, 0.1% TFA in water, and solvent B, acetonitrile, degassed by helium bubble and sonication, respectively. HRMS was done on a Bruker microTOF II mass spectrometer using electrospray ionization (ESI-TOF) operating in the positive mode. Adducts within errors of ±10 ppm were reported.

#### 3-(Chloromethyl)-6,6-dimethyl-5,6-dihydroimidazo[2,1-*b*]thiazole (**6**)

4,4-dimethylimidazolidine-2-thione (500 mg, 3.84 mmol) was dissolved in DCM (12 mL), and 1,3-dichloroacetone (0.35 mL, 1 equiv) was added to it. This mixture was left to reflux for 2 h, after which the reaction was concentrated in vacuo and the intermediate was precipitated out by triturating with diethyl ether. This intermediate was filtered off, washed with diethyl ether, and suspended in diglyme (3.5 mL). This mixture was then heated at 140 °C for 2 h, solids were filtered off and washed with diethyl ether to give compound **6** as an HCl salt (0.62 g, 2.57 mmol, 67% yield, white solid). ^1^H NMR (400 MHz, DMSO-*d*_6_): δ 10.94 (s, 1H), 7.07 (s, 1H), 4.86 (s, 2H), 4.22 (s, 2H), 1.50 (s, 6H). ^13^C NMR (101 MHz, DMSO-*d*_6_): δ 168.36, 133.17, 111.08, 69.48, 57.84, 35.90, 27.44.

#### *tert*-Butyl ((1*R*,4*R*)-4-(3-Cyclohexylthioureido)cyclohexyl)carbamate (**7**)

*tert*-Butyl((1*R*,4*R*)-4-aminocyclohexyl)-carbamate (0.43 g, 2.0 mmol) and cyclohexyl isothiocyanate (0.567 mL, 4.0 mmol, 2 equiv) were taken up in DCM (10 mL), and the reaction mixture was left to stir at room temperature for 20 h. The mixture was then concentrated in vacuo and purified by column chromatography using 2% MeOH in DCM as the eluent. The product was isolated as a brownish oil (0.60 g, 1.7 mmol, 85% yield). ^1^H NMR (400 MHz, CDCl_3_): δ 5.74 (s, 1H), 5.49 (s, 1H), 4.46–4.39 (m, 1H), 4.03 (s, 1H), 3.73 (s, 1H), 3.55–3.28 (m, 1H), 2.14 (s, 2H), 2.07–1.91 (m, 4H), 1.76–1.67 (m, 2H), 1.65–1.57 (m, 1H), 1.42 (s, 9H), 1.40–1.31 (m, 2H), 1.33–1.14 (m, 8H).

#### *tert*-Butyl ((1*R*,4*R*)-4-(((*E*)-(Cyclohexylamino) (((6,6-dimethyl-5,6-dihydroimidazo [2,1-*b*]thiazol-3-yl)methyl)thio)methylene)amino)cyclohexyl)carbamate Hydrochloride Salt (**8**)

*tert*-Butyl ((1*R*,4*R*)-4-(3-cyclohexylthioureido)cyclohexyl)carbamate (0.1 g, 0.28 mmol) and 3-(chloromethyl)-6,6-dimethyl-5,6-dihydroimidazo[2,1-*b*]thiazole, hydrochloride salt (0.075 g, 0.3 mmol) in dry acetonitrile (3 mL) was heated at 80 °C over night. After cooling, the reaction mixture was evaporated to dryness. Purification was by reverse-phase silica gel chromatography eluting with a gradient of 10% to 90% acetonitrile in water. Yield 0.13 g, 0.25 mmol. LC–MS 522, M^+1^, rt 1.99 min. ^1^H NMR (400 MHz, DMSO-*d*_6_): δ 10.55 (s, 1H), 9.84–9.80 (m, 1H), 9.32–9.25 (m, 1H), 6.89 (s, 1H), 6.87–6.83 (m, 1H), 4.87 (s, 2H), 4.25 (s, 2H), 3.95–3.55 (m, 1H), 3.47–3.24 (m, 2H), 3.17 (s, 1H), 1.90–1.00 (m, 18H), 1.51 (s, 6H), 1.38 (s, 9H). ^13^C NMR (101 MHz, methanol-*d*_4_) 169.15, 161.70, 156.42, 131.79, 110.08, 78.68, 69.94, 58.21, 57.43, 56.13, 53.94, 53.00, 32.35, 31.40, 30.67, 30.08, 28.58, 27.35, 24.51.

#### (6,6-Dimethyl-5,6-dihydroimidazo[2,1-*b*]thiazol-3-yl)methyl (*E*)-*N*′-((1*R*,4*R*)-4-Aminocyclo-hexyl)-*N*-cyclohexylcarbamimidothioate Trifluoroacetate Salt (**9**)

Carbamate **8** (0.10 g, 0.20 mmol) was taken up in DCM (8.0 mL), and TFA (2.0 mL) was carefully added to it. The solution was stirred at room temperature for 3.5 h, after which TLC indicated full conversion. The solvents were removed in vacuo and the TFA was removed under high vacuum. The crude was purified by column chromatography, eluting with a gradient of 0–10% 7 N NH_3_ in MeOH in DCM as eluent, affording the desired product as a colorless oil (84 mg, 0.19 mmol, 99% yield). LC–MS; *m*/*z*: 422.4 [M + H]^+^. ^1^H NMR (400 MHz, MeOD-*d*_4_): δ 6.93 (s, 1H), 4.58 (s, 2H), 4.33 (s, 2H), 3.95–3.82 (m, 1H), 3.18–3.15 (m, 2H), 2.25–2.08 (m, 7H), 2.03–1.80 (m, 8H), 1.60–1.57 (m, 3H), 1.49 (s, 3H), 1.44–1.37 (m, 3H). ^13^C NMR (101 MHz, MeOD-*d*_4_): δ 170.51, 162.87, 162.52, 111.40, 71.26, 66.87, 64.51, 59.43, 58.96, 30.26, 30.15, 29.98, 29.88, 29.61, 28.02, 27.49, 26.76, 25.85.

#### (6,6-Dimethyl-5,6-dihydroimidazo[2,1-*b*]thiazol-3-yl)methyl (*E*)-*N*-Cyclohexyl-*N*′-((1*R*,4*R*)-4-(6-(2-(4-((*E*)-2-(5,5-difluoro-7-(thiophen-2-yl)-5*H*-4λ^4^,5λ^4^-dipyrrolo[1,2-*c*:2′,1′-*f*][1,3,2]diazaborinin-3-yl)vinyl)phenoxy)acetamido)hexanamido)cyclo-hexyl)carbamimidothioate (**10**)

2,5-Dioxopyrrolidin-1-yl (E)-6-(2-(4-(2-(5,5-difluoro-7-(thiophen-2-yl)-5*H*-4l4,5l4-dipyrrolo[1,2-*c*:2′,1′-*f*][1,3,2]diazaborinin-3-yl)vinyl)phenoxy)acetamido)hexanoate (BDP 630/650 X NHS ester) (1.5 × 10^–6^ mol, 1 mg) in anhydrous DMF (0.75 mL) was added to a mixture of (6,6-dimethyl-5,6-dihydroimidazo[2,1-*b*]thiazol-3-yl)methyl (*Z*)-*N*′-((1*R*,4*R*)-4-aminocyclohexyl)-*N*-cyclohexylcarbamimidothioate, trifluoroacetate salt (1.92 × 10^–6^ mol), and diisopropylethylamine (10 mg, 7.7 × 10^–5^ mol) in anhydrous DMF (0.75 mL). The blue solution was stored in the dark overnight. The solution was evaporated to dryness under high vacuum (temperature of water bath <35 °C). Purification was by reverse-phase semi-prep chromatography (20–95% MeCN in H_2_O, containing 0.1% formic acid). Product containing fractions were combined and lyophilized to give the product (0.9 mg, 9.3 × 10^–7^ mol, 62%) HRMS: C_50_H_62_BF_2_N_8_O_3_S_3_: M^+1^; found, 967.4169, theory: 967.4163. Purity of the compound was confirmed to be >95% by LC–MS.

#### 1-(6-(((1*R*,4*R*)-4-(((*Z*)-(Cyclohexylamino) (((6,6-dimethyl-5,6-dihydroimidazo[2,1-*b*]thiazol-3-yl)methyl)thio)methylene)amino)cyclohexyl)amino)-6-oxohexyl)-3,3-dimethyl-2-((1*E*,3*E*)-5-((*Z*)-1,3,3-trimethyl-5-sulfoindolin-2-ylidene)penta-1,3-dien-1-yl)-3*H*-indol-1-ium-5-sulfonate (**11**)

1-(6-((2,5-dioxopyrrolidin-1-yl)oxy)-6-oxohexyl)-3,3-dimethyl-2-((1*E*,3*E*)-5-((*Z*)-1,3,3-trimethyl-5-sulfoindolin-2-ylidene)penta-1,3-dien-1-yl)-3*H*-indol-1-ium-5-sulfonate (sulfo-cyanine5 NHS ester) (1.2 × 10^–6^ mol, 0.9 mg) in anhydrous DMF (0.75 mL) was added to a mixture of (6,6-dimethyl-5,6-dihydroimidazo[2,1-*b*]thiazol-3-yl)methyl (*Z*)-*N*′-((1*R*,4*R*)-4-aminocyclohexyl)-*N*-cyclohexylcarbamimidothioate, trifluoroacetate salt (1.7 × 10^–6^ mol), and diisopropylethylamine (10 mg, 7.7 × 10^–5^ mol) in anhydrous DMF (0.75 mL). The blue solution was stored in the dark overnight. The solution was evaporated to dryness under high vacuum (temperature of water bath <35 °C). Purification was by reverse-phase semi-prep chromatography (20–95% MeCN in H_2_O, containing 0.1% formic acid). Product containing fractions were combined and lyophilized to give the product (1.01 mg, 9.7 × 10^–7^ mol, 81%) HRMS: C_53_H_72_N_7_O_7_S_4_: M + 1; found, 1046.4337, theory: 1046.4371. Purity of the compound was confirmed to be >95% by LC–MS.

#### 4-Iodo-3-methylisoquinoline (**13**)

To a round-bottom flask containing glacial acetic acid (10 mL) were added 3-methylisoquinoline (0.29 g, 2.0 mmol) and NIS (0.50 g, 2.2 mmol, 1.1 equiv), and the reaction was stirred at 80 °C for 16 h. Upon completion (TLC), the reaction mixture was quenched with saturated aqueous NaHCO_3_ while keeping the round-bottom flask on ice. The product was extracted by DCM (3 × 100 mL), washed with brine, and dried over MgSO_4_. Column chromatography eluting with 5% EtOAc in DCM yielded the title compound as a light yellow crystalline solid (0.28 g, 1.03 mmol, 52% yield). ^1^H NMR (400 MHz, CDCl_3_): δ 9.03 (s, 1H), 8.08 (dd, *J* = 8.6, 1.1 Hz, 1H), 7.87 (d, *J* = 8.1 Hz, 1H), 7.75 (ddd, *J* = 8.4, 6.9, 1.3 Hz, 1H), 7.59 (ddd, *J* = 8.0, 6.9, 1.0 Hz, 1H), 2.99 (s, 3H).

#### *tert*-Butyl (3-((1*R*,5*R*)-9-Borabicyclo[3.3.1]nonan-9-yl)propyl)carbamate (**14**)

A round-bottom flask containing 9-BBN (7.0 mL, 0.5 M solution in THF) was cooled to 0 °C and *tert*-butyl *N*-allylcarbamate (20 mg, 3.2 mmol) was added to it. This mixture was stirred at 0 °C for 1.5 h, after which the reaction mixture was allowed to reach room temperature at which it was stirred for another 1.5 h. The hydroborated product was directly used in the next step without isolation or purification.

#### *tert*-Butyl (3-(3-Methylisoquinolin-4-yl)propyl)carbamate (**15**)

The hydroborated allyl carbamate (**14**) was transferred into a microwave vessel and to it were added isoquinoline **13** (0.69 g, 2.56 mmol, 0.8 equiv), KOH (2.5 mL, 1.0 M in H_2_O), and Pd(PPh_3_)_4_ (0.37 g, 0.1 equiv). The reaction was irradiated at 80 °C for 1 h. The reaction mixture was poured onto brine and extracted with EtOAc (2 × 50 mL). The organics were combined, dried over MgSO_4_, and concentrated in vacuo. The title compound was purified by column chromatography, eluting with 20–100% EtOAc in pet ether, yielding a light yellow oil (0.21 g, 0.7 mmol 27% yield over two steps). ^1^H NMR (400 MHz, CDCl_3_) 9.05 (s, 1H), 7.93 (td, *J* = 8.6, 7.9, 1.0 Hz, 2H), 7.68 (ddd, *J* = 8.5, 6.8, 1.4 Hz, 1H), 7.51 (ddd, *J* = 8.0, 6.8, 1.1 Hz, 1H), 4.81 (s, 1H), 3.33–3.23 (m, 2H), 3.13–2.99 (m, 2H), 2.71 (s, 3H), 1.95–1.76 (m, 2H), 1.46 (s, 9H). ^13^C NMR (101 MHz, CDCl_3_): δ 156.07, 150.07, 148.97, 135.03, 130.29, 128.28, 127.58, 127.31, 125.75, 122.40, 40.77, 32.87, 30.25, 28.42, 25.41, 22.16.

#### *tert*-Butyl (3-(3-(Bromomethyl)isoquinolin-4-yl)propyl)carbamate (**16**)

A round-bottom flask containing DCE (10 mL) was charged with methylisoquinoline **15** (0.21 g, 0.7 mmol), NBS (0.17 g, 0.91 mmol, 1.3 equiv), and AIBN (23 mg, 0.14 mmol, 0.2 equiv). The reaction mixture was stirred under a N_2_ atmosphere at 80 °C for 3 h, after which LC–MS showed ∼70% conversion to the product, the solvents were removed in vacuo and the crude product was, due to its apparent instability, directly used in the next step.

#### *tert*-Butyl (*S*)-(3-(3-((Methyl(5,6,7,8-tetrahydroquinolin-8-yl)amino)methyl)isoquinolin-4-yl)propyl)carbamate (**17**)

The crude bromomethyl compound **16** was taken up in acetonitrile (10 mL), and MeCN was added to it. **12** (0.10 g, 0.8 equiv) and DIPEA (0.5 mL, 4 equiv) were added, and the reaction was stirred for 16 h, after which TLC samples showed completion of the reaction. The title compound was purified by column chromatography, eluting first with EtOAc in pet ether (1:1), followed by 2–5% 7 N NH_3_ in MeOH in DCM, yielding the title compound as a brown solid (55 mg, 0.12 mmol, 21% over 2 steps). ^1^H NMR (400 MHz, CDCl_3_): δ 9.07 (s, 1H), 8.55 (dd, *J* = 4.8, 1.7 Hz, 1H), 7.99 (dt, *J* = 8.6, 0.9 Hz, 1H), 7.92 (dt, *J* = 8.1, 1.0 Hz, 1H), 7.67 (ddd, *J* = 8.5, 6.8, 1.4 Hz, 1H), 7.53 (ddd, *J* = 7.9, 6.8, 1.0 Hz, 1H), 7.36 (ddt, *J* = 7.8, 1.9, 1.0 Hz, 1H), 7.06 (ddd, *J* = 7.7, 4.7, 0.6 Hz, 1H), 4.86 (s, 1H), 4.16–4.00 (m, 3H), 3.26 (t, *J* = 8.0 Hz, 2H), 2.93–2.62 (m, 4H), 2.27 (s, 3H), 2.12 (dtd, *J* = 8.8, 5.5, 2.6 Hz, 2H), 2.08–1.98 (m, 1H), 1.95–1.75 (m, 2H), 1.68 (tdd, *J* = 13.4, 9.9, 5.2 Hz, 1H), 1.47 (s, 9H).

#### (*S*)-*N*-((4-(3-Aminopropyl)isoquinolin-3-yl)methyl)-*N*-methyl-5,6,7,8-tetrahydroquinolin-8-amine (**3**)

*N*-boc-protected **17** (55 mg, 0.12 mmol) was taken up in DCM (8 mL) and TFA (2 mL) was added to it. The resulting solution was stirred for 1 h, after which TLC showed full conversion. The reaction mixture was concentrated in vacuo and residual TFA was removed under high vacuum. Column chromatography, eluting with 5% 7 N NH_3_ in MeOH in DCM afforded the congener as a colorless oil (21 mg, 0.06 mmol, 49% yield). ^1^H NMR (400 MHz, MeOD-*d*_4_): δ 9.02 (s, 1H), 8.44 (dd, *J* = 4.8, 1.6 Hz, 1H), 8.13 (dd, *J* = 8.7, 1.0 Hz, 1H), 8.05 (dt, *J* = 8.1, 1.0 Hz, 1H), 7.79 (ddd, *J* = 8.4, 6.8, 1.3 Hz, 1H), 7.64 (ddd, *J* = 8.0, 6.8, 1.0 Hz, 1H), 7.55 (ddd, *J* = 7.8, 1.8, 0.9 Hz, 1H), 7.22 (dd, *J* = 7.7, 4.8 Hz, 1H), 4.08–3.90 (m, 3H), 3.29–3.15 (m, 2H), 2.90 (ddd, *J* = 15.4, 9.5, 5.3 Hz, 1H), 2.82–2.67 (m, 3H), 2.29–1.96 (m, 5H), 1.96–1.52 (m, 2H). ^13^C NMR (101 MHz, MeOD-*d*_4_): δ 158.02, 150.56, 149.88, 147.40, 138.87, 136.79, 136.45, 133.04, 132.07, 129.48, 128.21, 124.37, 123.58, 64.89, 58.93, 41.88, 38.64, 33.20, 29.87, 25.37, 23.96, 21.79.

#### (*S*,*E*)-6-(2-(4-(2-(5,5-Difluoro-7-(thiophen-2-yl)-5*H*-4λ^4^,5λ^4^-dipyrrolo[1,2-*c*:2′,1′-*f*][1,3,2]diazaborinin-3-yl)vinyl)phenoxy)acetamido)-*N*-(3-(3-((methyl(5,6,7,8-tetrahydroquinolin-8-yl)amino)methyl)isoquinolin-4-yl)propyl)hexanamide (**18a**)

BODIPY 630/650-X succinimide ester (1.0 mg, 1.5 μmol) was dissolved in acetonitrile (1.0 mL), and **3** (1.0 mg, 2.85 μmol, 1.9 equiv) and DIPEA (2.0 μL, 6.0 μmol, 4 equiv) were added to it. This mixture was left to stir in the dark for 4 h. LC–MS indicated full consumption of the BODIPY dye, so solvents were evaporated in vacuo and the title compound purified via preparative RP-HPLC (5–95% solvent B, 30 min), which after lyophilization yielded blue solids (1.3 mg, 0.77 μmol, 51% yield). HRMS (ESI-TOF) calcd for C_52_H_55_BF_2_N_7_O_3_S [M + H]^+^, 906.4143; found, 906.4190.

#### (*S*)-6-(3-(5,5-Difluoro-7,9-dimethyl-5*H*-4λ^4^,5λ^4^-dipyrrolo[1,2-*c*:2′,1′-*f*][1,3,2]diazaborinin-2-yl)propanamido)-*N*-(3-(3-((methyl(5,6,7,8-tetrahydroquinolin-8-yl)amino)methyl)isoquinolin-4-yl)propyl)hexanamide (**18b**)

BODIPY FL-X succinimidyl ester (1.2 mg, 3 μmol) was dissolved in acetonitrile (1.0 mL), and compound **3** (1.0 mg, 2.8 μmol, 0.93 equiv) and DIPEA (2.0 μL, 12 μmol, 4 eq.) were added to it, and the reaction was stirred in the dark for 72 h. LC–MS indicated full consumption of the BODIPY dye, so solvents were removed in vacuo and the title compound was purified by preparative RP-HPLC (5–95% solvent B, 30 min), which after lyophilization yielded orange solids (1.0 mg, 1.65 μmol, 55%). HRMS (ESI-TOF) calcd for C_43_H_53_BF_2_N_7_O_2_ [M + H]^+^, 748.4316; found, 748.4322.

#### 4-(1,3-Dioxoisoindolin-2-yl)butanal (**19**)

Step one: 4-amino-1-butanol (0.2 mL, 2.2 mmol) was taken up in toluene (25 mL), and phthalic anhydride (0.32 g, 2.2 mmol, 1 equiv) and 4 Å molecular sieves were added to it. The reaction mixture was stirred at a reflux for 3 h, after which TLC samples indicated full conversion. The solvents were removed in vacuo and the phthalimide was purified by column chromatography, eluting with 0–2% MeOH in CHCl_3_. Step two: to a stirring solution of DMP (0.57 g, 1.35 mmol, 1.5 equiv) in DCM (15 mL) at 0 °C was added in dropwise fashion the intermediate phthalimide (0.20 g, 0.9 mmol, in 5 mL DCM). The reaction mixture was allowed to reach room temperature at which it was maintained until completion (TLC). The reaction mixture was then concentrated to a third of its original volume and diethyl ether was added. This was then washed with saturated aqueous solutions of Na_2_S_2_O_3_ and NaHCO_3_. The organic solvent was dried over MgSO_4_ and finally removed in vacuo, affording the title compound as a colorless oil (0.14 g, 0.66 mmol, 40% yield over two steps). ^1^H NMR (400 MHz, CDCl_3_): δ 9.77 (d, *J* = 1.2 Hz, 1H), 7.84 (dd, *J* = 5.4, 3.1 Hz, 2H), 7.72 (dd, *J* = 5.4, 3.1 Hz, 2H), 3.74 (t, *J* = 6.8 Hz, 2H), 2.54 (t, *J* = 7.3 Hz, 2H), 2.02 (p, *J* = 7.0 Hz, 2H).

#### 2-(4-(((*S*)-1-(4-Methoxyphenyl)ethyl) ((*S*)-5,6,7,8-tetrahydroquinolin-8-yl)amino)butyl)iso-indoline-1,3-dione (**20**)

To a suspension of NaBH(OAc)_3_ (0.16 g, 2 equiv) in DCM (15 mL) were added aldehyde **19** (0.08 g, 0.37 mmol) and amine **25** (0.10 g, 0.37 mmol, 1 equiv), and the reaction was stirred under a N_2_ atmosphere overnight. Upon completion of the reaction (TLC), the reaction mixture was diluted with DCM and added to a vigorously stirring solution of saturated aqueous NaHCO_3_. After 15 min, the layers were separated, the aqueous washed with DCM, and the combined organics washed with brine. After drying over MgSO_4_, the solvents were evaporated and the title compound was purified by column chromatography, eluting with 2–10% MeOH in DCM, affording a white solid (0.12 g, 0.25 mmol, 69% yield). ^1^H NMR (400 MHz, CDCl_3_): δ 8.37 (s, 1H), 7.80 (dt, *J* = 5.6, 2.9 Hz, 2H), 7.70 (dq, *J* = 7.1, 4.2 Hz, 2H), 7.39 (s, 2H), 7.16 (d, *J* = 7.6 Hz, 1H), 6.94–6.80 (m, 3H), 4.48 (br s, 1H) 3.95 (br s, 1H), 3.77 (s, 3H), 3.48–3.42 (m, 3H), 2.91–2.70 (m, 1H), 2.61 (dt, *J* = 16.8, 5.1 Hz, 1H), 2.57–2.38 (m, 2H), 2.17–1.70 (m, 4H), 1.64–1.51 (m, 1H), 1.45–1.25 (m, 5H), 1.01–0.91 (m, 1H).

#### (*S*)-2-(4-((5,6,7,8-Tetrahydroquinolin-8-yl)amino)butyl)isoindoline-1,3-dione (**21**)

Compound **20** (0.12 g, 0.24 mmol) was taken up in DCM (10 mL) and to it was carefully added TFA (10 mL). The resulting solution was stirred at room temperature for 30 min, after which LC–MS indicated full conversion. The reaction mixture was concentrated in vacuo and residual TFA was removed under high vacuum. For storage purposes, the product was kept as an oxalate salt, by dissolving the title compound in IPA (10 mL), and adding oxalic acid (32 mg, 1.3 equiv). This was stirred for 10 min and solvents evaporated yielding the title compound as an oxalate salt (0.11 g, 0.24 mmol, quant. colorless oil). ^1^H NMR (400 MHz, DMSO-*d*_6_): δ 8.87 (s, 2H), 8.48 (d, *J* = 4.7 Hz, 1H), 7.94–7.79 (m, 4H), 7.66 (d, *J* = 7.8 Hz, 1H), 7.37 (dd, *J* = 7.8, 4.7 Hz, 1H), 3.73–3.65 (m, 1H), 3.61 (t, *J* = 8.0 Hz, 2H), 3.02–2.04 (m, 2H), 2.81–2.78 (m, 2H), 2.32 (dd, *J* = 11.4, 4.9 Hz, 1H), 1.98 (dd, *J* = 11.3, 5.4 Hz, 1H), 1.90–1.58 (m, 6H). ^13^C NMR (101 MHz, DMSO-*d*_6_): δ 168.46, 161.44, 158.78, 158.43, 151.10, 147.15, 138.31, 134.94, 133.93, 132.07, 124.15, 123.53, 62.49, 56.38, 44.17, 37.32, 27.60, 25.95, 25.63, 25.13, 23.59, 19.83.

#### *tert*-Butyl 2-(Bromomethyl)-1*H*-benzo[*d*]imidazole-1-carboxylate (**22**)

Step one: 2-methylbenzimidazole (1.0 g, 0.76 mmol) was taken up in DCM (25 mL) and TEA (1.1 mL, 0.8 mmol, 1.05 equiv), DMAP (46 mg, 0.04 mmol, 0.05 equiv), and boc anhydride (2.5 g, 1.52 mmol, 2 equiv) were added to it. The resulting solution was stirred for 5 min, after which it was concentrated in vacuo. The residue was taken up in H_2_O (20 mL) and stirred for 15 min. The solids were filtered off, washed with cold H_2_O, and subsequently dried. Step two: the *N*-boc-protected imidazole (1.6 g, 7.2 mmol) was taken up in DCE (35 mL), and NBS (1.4 g, 7.9 mmol, 1.1 equiv) and AIBN (0.3 g, 1.8 mmol, 0.25 equiv) were added to it, and the reaction was stirred at 95 °C for 6 h. The reaction mixture was then concentrated in vacuo and purified by column chromatography, eluting with 5–10% EtOAc in pet ether, providing the title compound as a white solid (1.47 g, 4.7 mmol, 63% yield over two steps). ^1^H NMR (400 MHz, CDCl_3_): δ 8.04–7.93 (m, 1H), 7.80–7.68 (m, 1H), 7.45–7.31 (m, 2H), 5.07 (s, 0.5H), 4.96 (s, 1.5H), 1.74 (s, 9H). ^13^C NMR (101 MHz, CDCl_3_): δ 150.55, 141.91, 133.49, 125.87, 125.83, 124.81, 124.77, 120.62, 120.55, 115.31, 115.26, 86.65, 28.10, 25.31, 23.60.

#### *tert*-Butyl (*S*)-2-(((4-(1,3-Dioxoisoindolin-2-yl)butyl) (5,6,7,8-tetrahydroquinolin-8-yl)amino)methyl)-1*H*-benzo[*d*]imidazole-1-carboxylate (**23**)

A microwave vessel was charged with **21** (0.08 g, 0.17 mmol), **22** (0.05 g, 0.16 mmol, 0.95 equiv), TEA (0.1 mL, 0.51 mmol, 3 equiv), KI (5 mg, 0.03 mmol, 0.2 equiv), and acetonitrile (3 mL). This mixture was irradiated at 80 °C for 1 h, after which the reaction mixture was concentrated onto silica and purified by column chromatography eluting with 0–10% MeOH in DCM. The title compound was isolated as a brownish oil (25 mg, 0.04 mmol, 27% yield). ^1^H NMR (400 MHz, CDCl_3_): δ 8.58 (d, *J* = 4.8 Hz, 1H), 7.75 (dd, *J* = 5.5, 3.1 Hz, 2H), 7.65 (dd, *J* = 5.4, 3.1 Hz, 2H), 7.63–7.54 (m, 2H), 7.39 (d, *J* = 7.7 Hz, 1H), 7.18 (dd, *J* = 6.0, 3.1 Hz, 2H), 7.12 (dd, *J* = 7.7, 4.7 Hz, 1H), 4.15–3.96 (m, 3H), 3.52 (t, *J* = 7.1 Hz, 2H), 2.83 (ddt, *J* = 14.7, 8.8, 4.6 Hz, 1H), 2.78–2.65 (m, 2H), 2.65–2.53 (m, 1H), 2.29–2.14 (m, 1H), 2.09–1.97 (m, 1H), 1.96–1.85 (m, 1H), 1.72 (s, 9H), 1.70–1.52 (m, 3H), 1.52–1.32 (m, 2H). ^13^C NMR (101 MHz, CDCl_3_): δ 168.48, 157.59, 156.55, 146.87, 137.34, 134.61, 133.90, 132.14, 123.22, 122.25, 121.61, 61.63, 49.90, 49.75, 37.68, 29.33, 28.21, 26.09, 25.77, 23.69, 21.59.

#### (*S*)-*N*1-((1*H*-Benzo[*d*]imidazole-2-yl)methyl)-*N*1-(5,6,7,8-tetrahydroquinolin-8-yl)butane-1,4-diamine (**2**)

A microwave vessel was charged with **23** (13 mg, 0.03 mmol), hydrazine (20 μL, hydrazine (80% in H_2_O, 20 μL, 0.5 mmol, 16.7 equiv), and ethanol (2.0 mL). The resulting solution was irradiated at 80 °C for 1 h. The solvents were removed in vacuo and the product was purified by column chromatography, eluting with 8% 7 N NH_3_ in MeOH in DCM, yielding the title compound as a brown solid (8 mg, 0.02 mmol, 84% yield). ^1^H NMR (400 MHz, CDCl_3_): δ 8.61 (d, *J* = 8.0 Hz, 1H), 7.61 (dd, *J* = 6.0, 3.3 Hz, 2H), 7.44 (d, *J* = 7.6 Hz, 1H), 7.21 (dd, *J* = 6.0, 3.2 Hz, 2H), 7.16 (dd, *J* = 7.7, 4.8 Hz, 1H), 4.16–3.98 (m, 3H), 2.96–2.81 (m, 1H), 2.81–2.66 (m, 2H), 2.66–2.49 (m, 3H), 2.30–2.17 (m, 1H), 2.14–2.01 (m, 1H), 1.93 (tdd, *J* = 12.8, 10.0, 2.8 Hz, 1H), 1.81–1.64 (m, 1H), 1.57–1.32 (m, 4H). ^13^C NMR (101 MHz, CDCl_3_): δ 157.61, 156.52, 146.81, 137.46, 134.75, 122.28, 121.68, 61.89, 53.56, 50.62, 49.62, 41.78, 31.01, 29.37, 26.04, 23.49, 21.53.

#### (*S*,*E*)-*N*-(4-(((1*H*-Benzo[*d*]imidazole-2-yl)methyl) (5,6,7,8-tetrahydroquinolin-8-yl)amino)butyl)-6-(2-(4-(2-(5,5-difluoro-7-(thiophen-2-yl)-5*H*-4λ4,5λ4-dipyrrolo [1,2-*c*:2′,1′-*f*][1,3,2]diazaborinin-3-yl)vinyl)phenoxy)acetamido)hexanamide (**24**)

BODIPY 630/650-X succinimide ester (1.2 mg, 1.8 μmol) was dissolved in acetonitrile (1.0 mL), and to it were added **2** (1.0 mg, 2.9 μmol, 1.6 equiv) and DIPEA (2.0 μL, 7.2 μmol, 4 equiv). This mixture was left to stir in the dark for 16 h. LC–MS indicated full conversion of the BODIPY dye to the fluorescent conjugate, so solvents were removed in vacuo after which the crude was purified by preparative RP-HPLC (5–95% solvent B, 30 min), which after lyophilization yielded the title compound as a dark blue solid (0.9 mg, 1.0 μmol, 53% yield). Purity of the compound was confirmed to be >95% by LC–MS. HRMS (ESI-TOF) calcd for C_50_H_53_BF_2_N_8_O_3_S [M + H]^+^, 895.4101; found, 895.4036.

### Molecular Modeling of CXCR4 Receptor Antagonist AMD070

Docking of AMD070 to the high-resolution CXCR4 crystal structure was performed using Schrodinger software suite (release 2018–3). The 2.5 Å resolution CXCR4 crystal structure was imported from the Protein Data Bank (PDB: 3ODU) and was prepared by the protein preparation wizard. This involved the removal of water molecules, cocrystallized head groups, with the exclusion of the cocrystallized ligand ZM241385, and the addition of hydrogen atoms. The H-bonding network was optimized using PROPKA at pH = 7, and ultimately, the protein structure was energy minimized by using the OPLS3 force field. The receptor grid was generated using the grid preparation tool, and the 2D molecular structures of the ligands were converted into 3D structures using the LigPrep tool. Compounds were docked using the glide docking tool with standard settings and enhanced planarity and Epik calculated protonation states at pH 6.8. Binding poses were generated, visually inspected, and ranked according to their GlideScore. Only docking poses with GlideScores lower than −7 were considered. The final pose was then exported as an image using the Schrödinger Suite.

### Pharmacology: Materials and Methods

Cell culture reagents were purchased from Sigma Chemicals (Pool, Dorset, UK) except fetal calf serum (FCS), which was provided by PAA Laboratories (Teddington, Middlesex, UK). CXCL12 was purchased from PeproTech, London, UK and Alexa Fluor 647-labeled CXCL12 was purchased from Almac, Edinburgh, UK. IT1t was purchased from Tocris Bioscience, Bristol, UK and AMD3100 was purchased from Sigma-Adrich, Gillingham, UK. Furimazine was purchased from Promega Corporation (Southampton, UK).

### Cell Culture

Cell line generation: the HEK293G-SNAP-CXCR4 clonal stable cell line was generated through the transfection of the SNAP-CXCR4 construct (Cisbio, Codolet, France) using lipofectamine (Thermo Fisher Scientific, Waltham, MA) according to manufacturer’s instructions. Briefly, HEK293 GloSensor cells (HEK293G) (Promega, Wisconsin, USA) were transfected with SNAP-CXCR4 at 300 ng/well in a six-well plate. The following day, plasmid expression was selected for and maintained in growth media in the presence of G418 (Sigma-Aldrich). Clonal lines were generated and screened for high expression following labeling with 0.5 μM SNAP-Surface Alexa Fluor 488 (New England Biolabs, Hitchin UK) for 30 min at 37 °C. Following clonal line generation, G418 use was discontinued. The HEK293G-NL-CXCR4 cell line was created as described by White et al.^40^

All cell lines were cultured in Dulbecco’s modified Eagle’s medium (DMEM; D6429, Sigma-Aldrich), supplemented with 10% FCS. Cells were grown in T75 tissue culture flasks (75 cm^2^) in a humidified atmosphere at 37 °C, in a humidified atmosphere of 95% air and 5% CO_2_. Cells were left to grow up to 80% confluence prior to passaging to prevent loss of protein expression and cell detachment. The growth medium (DMEM) was aspirated and cells gently washed with 5 mL of phosphate-buffered saline (PBS), followed by incubation with trypsin (1 mL) for 5 min. The cells were then collected and centrifuged for 5 min at 1000 rpm, with the resulting cell pellet then gently resuspended in 10 mL of growth medium and transferred to new T75 flasks at appropriate dilutions (typically 1:5 or 1:20 split ratios). For the preparation of HEK293G-NLuc-CXCR4 membranes, cells were cultured in 500 cm^2^ trays and left to grow to 90% confluency. The growth medium was aspirated and cells were gently washed with 10 mL of PBS to remove any residual medium. 20 mL of fresh PBS was then added and cells were removed from the dish using a cell scraper. The cells were then transferred into a falcon tube and centrifuged at 1500 rpm for 10 min at 4 °C. The supernatant was discarded, cells resuspended in 15 mL of PBS and then homogenized in 10 × 2 s bursts at 15,000 rpm using an Ultra-Turrax dispersing instrument (a mechanical disruption device with rotating blades). Homogenates were then centrifuged at 1500*g* for 20 min at 4 °C to remove any unbroken cells. The resulting supernatant was transferred to a high speed centrifuge tube and centrifuged for 30 min at 41,415*g* at 4 °C to obtain membrane pellets. Pellets were resuspended in ice cold PBS and homogenized using a motor-driven pestle with a glass/Teflon homogenizer.

Quantification of the protein content of membranes was performed using a Pierce BCA Protein Assay Kit (Thermo Fisher Scientific) and membranes stored at −80 °C prior to use in assays.

### NanoBRET Saturation Assays

In white clear-bottom 96-well plates (655089; Grenier Bio-One or white opaque bottom 96 well plates), membranes generated from HEK293G Nluc-CXCR4 expressing cells, were diluted to 10 μg per well in HEPES-buffered saline solution containing 0.2% BSA (HBSS; 145 mM NaCl, 5 mM KCl, 1.3 mM CaCl_2_, 1 mM MgSO_4_, 10 mM HEPES, 2 mM sodium pyruvate, 1.5 mM NaHCO_3_, 10 mM d-glucose, pH 7.45) and added to each well as a 20 μL aliquot. To a total well volume of 50 μL, competing unlabeled ligand (AMD3100, final concentration of 10 μM), and 20 μL of relevant fluorescent ligand (0–1000 nM in well concentration) were added to appropriate wells, all diluted in HBSS/0.2% BSA. The plates were incubated in the dark for 1 h at 37 °C without supplemental CO_2_. The NLuc substrate furimazine (Promega Corporation) was then added (5 μL per well, 10 μM final concentration) and plates were immediately read using the PHERAstar FS plate reader (BMG Labtech, UK) at room temperature. For the red fluorescing ligands, NLuc and BODIPY 630/650 emissions were simultaneously collected using a 460 nm (80 nm band pass filter) and >610 nm longpass filter, respectively. For green fluorescing ligands, NLuc and BODIPY-FL-X emissions were simultaneously measured using a 475 nm (30 nm band pass filter) and 535 nm (30 nm band pass filter). The resulting raw BRET ratio was calculated by dividing fluorescence emissions (>610 nm emission for red ligands or the 535 nm emission for green ligands) by NLuc emissions.

#### NanoBRET Competition Binding Assays

In white clear-bottom 96-well plates or white opaque bottom 96-well plates, membranes expressing NLuc-CXCR4 were diluted to 10 μg per well in 20 μL of HBSS containing 0.2% BSA. To this, 10 μL of either compound **9b** or compound **16b** (in HBSS, final concentration of 100 and 250 nM, respectively) were added, alongside 20 μL of HBSS containing increasing concentrations of CXCL12 (0–1.0 μM concentration range) or the small molecules AMD3100 or IT1t (concentration range of 0–10.0 μM). Plates were then incubated in the dark for 2 h at 37 °C in the absence of CO_2_. The NLuc substrate furimazine (Promega Corporation) was then added (5 μL per well, 10 μM final concentration) and the plates were read using the PHERAstar FS plate reader (BMG Labtech, UK) at room temperature. The filtered light from each well was simultaneously measured using a 460 nm (80 nm band-pass filter) for NLuc emission and >610 nm longpass filter for fluorescence emission of BODIPY 630/650-X coupled ligands. For BODIPY-FL-X coupled ligands, emissions were simultaneously measured at 475 nm (30 nm band-pass filter; NLuc emission) and 535 nm (30 nm bandpass filter; BODIPY FL-X emission). The resulting raw BRET ratio was calculated by dividing the >610 nm emission (for red ligands) or the 535 nm emission (for green ligands) by the emissions measured for NLuc.

#### Data Analysis

All of the data generated was analyzed using Prism 7 software (GraphPad Software, San Diego, USA).

Saturation-binding curves were simultaneously fitted to obtain the total and non-specific components using the following equation
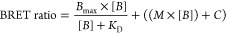
where *B*_max_ is the maximal level of specific binding, [*B*] is the concentration of the fluorescent ligand in nM, *K*_D_ is the equilibrium dissociation constant in nM, *M* is the slope of the linear non-specific binding component, and *C* is the *y*-axis intercept. Where the non-specific binding curve was linear, background and non-specific binding components were shared across all data sets with non-specific binding constrained to be greater than 0. When the non-specific binding curves showed saturable binding, total and non-specific binding curves were both fitted to the above equation with only background BRET shared between the data sets. Competition NanoBRET data were fitted using a one-site sigmoidal response curve given by the following equation
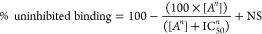
where [*A*] is the concentration of competing drug, NS is the non-specific binding, *n* is the Hill coefficient, and IC_50_ is the concentration of ligand required to inhibit 50% of the specific binding of the fluorescent ligand. The IC_50_ values from competition-binding curves were used to calculate the *K*_i_ of the unlabeled ligands using the Cheng–Prusoff equation
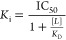
where [*L*] is the concentration of fluorescent ligand in nM, and *K*_D_ is the dissociation constant of the fluorescent ligand in nM. The *K*_D_ values used were obtained from the saturation binding experiments.

#### Confocal Microscopy

HEK293G-SNAP-CXCR4 cells were seeded onto poly-d-lysine-coated (10 μg/mL) 8-well Nunc Lab-tek chambered coverglass (no. 1.0 borosilicate glass bottom) in DMEM supplemented with 10% FCS at a density of 30,000 cells per well. The following day, media were replaced with fresh media supplemented with 10% FCS containing 0.5 μM SNAP-Surface Alexa Fluor 488 (New England Biolabs, Hitchin UK) for 30 min at 37 °C. Cells were then washed in warm HBSS (supplemented with 0.2% BSA) before pre-incubation in the absence or presence of 10 μM AMD3100 for 30 min at 37 °C in a volume of 180 μL HBSS (+0.2% BSA). Fluorescent ligand was then added (20 μL) to each well to achieve a final concentration of 10–50 nM, incubated for 30 min at 37 °C before being allowed to cool to 24 °C and imaged.

Cells were imaged on a Zeiss LSM880 with a Zeiss Axio Observer Z1 stand (Carl Zeiss, Germany) using a 40× C-apochromat NA1.2 water immersion objective. Excitation was via a 488 nm argon (to image SNAP-Surface Alexa Fluor 488) and 633 nm helium-neon (to image fluorescent ligands) laser lines with a 488/561/633 multibeam splitter and emission collected using a 493–628 nm band pass or 638–737 nm band pass. The pinhole was set at 1 Airy unit for the longer wavelength and laser power and gain and offset settings kept constant within the experiment to allow comparison. Equatorial plane images were made and four images were captured per condition per experiment using ZEN 2012 software (Carl Zeiss, Germany). Imaging of each ligand was performed at least three times.

## Supporting Information Available

Molecular formula strings (CSV)
Supporting Information S1 (LCMS traces of **10**, **11**, **18a**, **18b**, and **24**) (PDF)

## Abbreviations

BODIPY: boron dipyrromethene
BRET: bioluminescence resonance energy transfer
BSA: bovine serum albumin
cAMP: cyclic adenosine monophosphate
CXCL12: C–X–C chemokine ligand type 12
CXCR4: C–X–C chemokine receptor type 4
DMF: dimethylformamide
HEK293G: human embryonic kidney cells expressing a GloSensor biosensor
HIV-1: human immunodeficiency virus
LC–MS: liquid chromatography/mass spectrometry
NLuc: NLuciferase
NMR: nuclear magnetic resonance
PBS: phosphate-buffered saline
RP-HPLC: reverse-phase high-performance liquid chromatography
SAR: structure–activity relationship
SDF: stromal cell-derived factor 1
sulfo CY5: sulfo-cyanine 5
TOF ES^+^: positive electrospray ionization time-of-flight
