# Supplementary material for: Small-Molecule Fluorescent Ligands for the CXCR4 Chemokine Receptor
Source: J Med Chem. 2023 Mar 21;66(7):5208–22. doi: 10.1021/acs.jmedchem.3c00151 (PMC10108349; doi:10.1021/acs.jmedchem.3c00151)

S1: Supporting Information

Small molecule fluorescent ligands for the CXCR4 chemokine receptor

*Sebastian Dekkers,[1] Birgit Caspar,[2,3] Joëlle Goulding,[2,3] Nicholas D. Kindon [1], Laura E. Kilpatrick,[1,2] Leigh A Stoddart, [2,3] Stephen J. Briddon [2,3] Barrie Kellam,[1,2] Stephen J. Hill,[2,3] Michael J. Stocks[1]**

[1]Biodiscovery Institute, School of Pharmacy, University of Nottingham, Nottingham, NG7 2RD, United Kingdom

[2]Centre of Membrane Proteins and Receptors, University of Birmingham and University of Nottingham, The Midlands, NG7 2UH, United Kingdom.

[3]Division of Physiology, Pharmacology & Neuroscience, Medical School, University of Nottingham, Nottingham, NG7 2UH, UK.

*Corresponding Author: michael.stocks@nottingham.ac.uk

S2 LCMS purity trace compound **10**

S3 LCMS purity trace compound **11**

S4 LCMS purity trace compound **18a**

S5 LCMS purity trace compound **18b**

S6 LCMS purity trace compound **24**

LCMS purity trace compound **10**

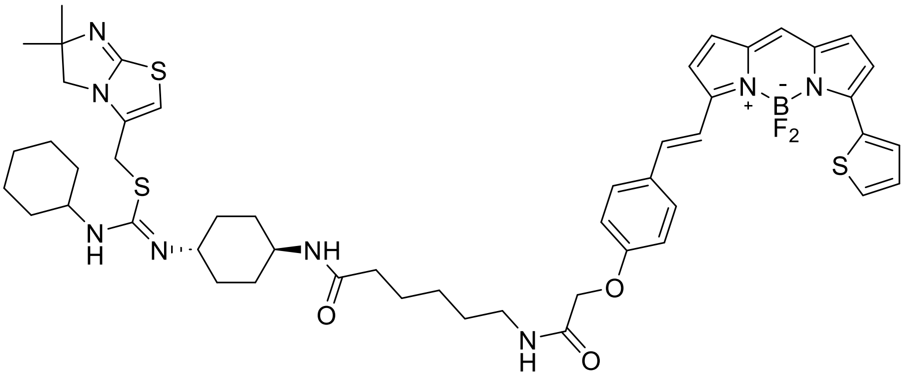

(6,6-Dimethyl-5,6-dihydroimidazo[2,1-b]thiazol-3-yl)methyl (E)-N-cyclohexyl-N'-((1r,4r)-4-(6-(2-(4-((E)-2-(5,5-difluoro-7-(thiophen-2-yl)-5H-4λ^{4},5λ^{4}-dipyrrolo[1,2-c:2',1'-f][1,3,2]diazaborinin-3-yl)vinyl)phenoxy)acetamido)hexanamido)cyclo-hexyl)carbamimidothioate

$C_{50}H_{62}BF_{2}N_{8}O_{3}S_{3}$: $[M+H]^{+}$ found: 967.4

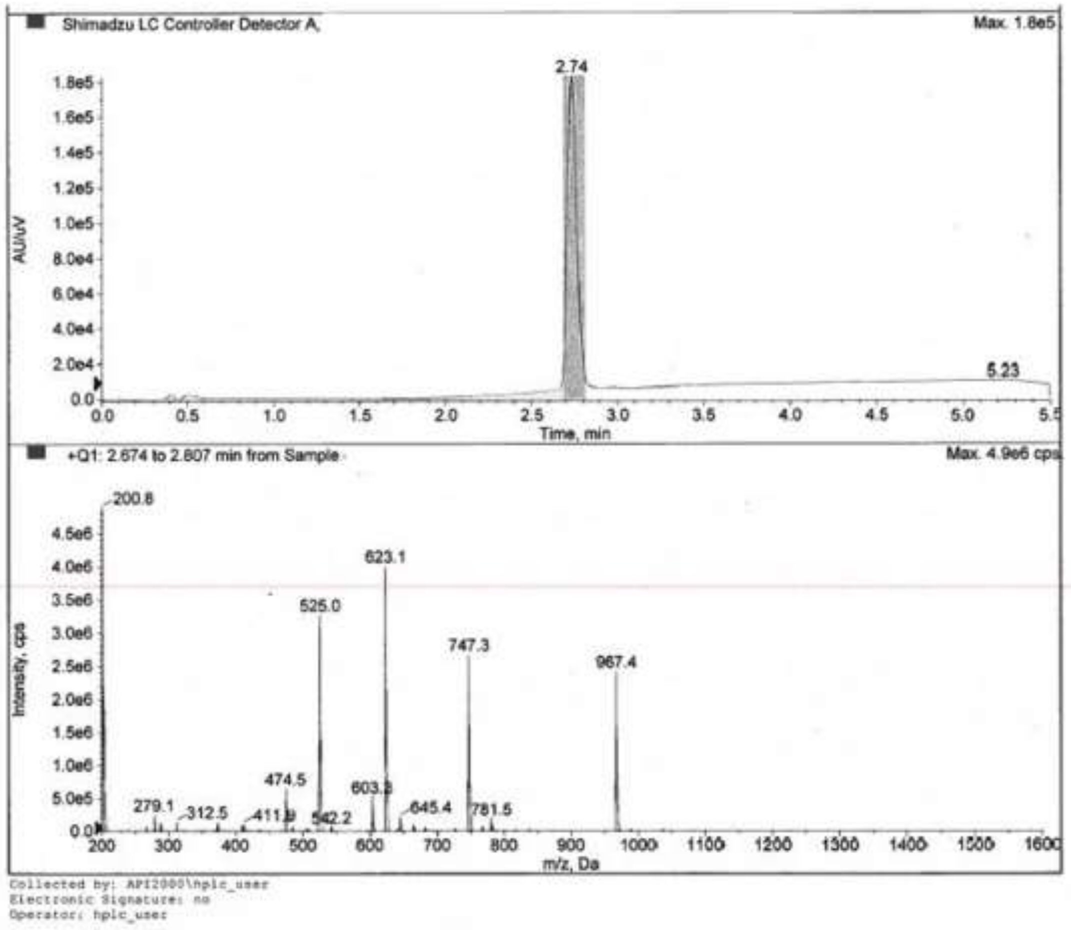

LCMS purity trace compound **11**

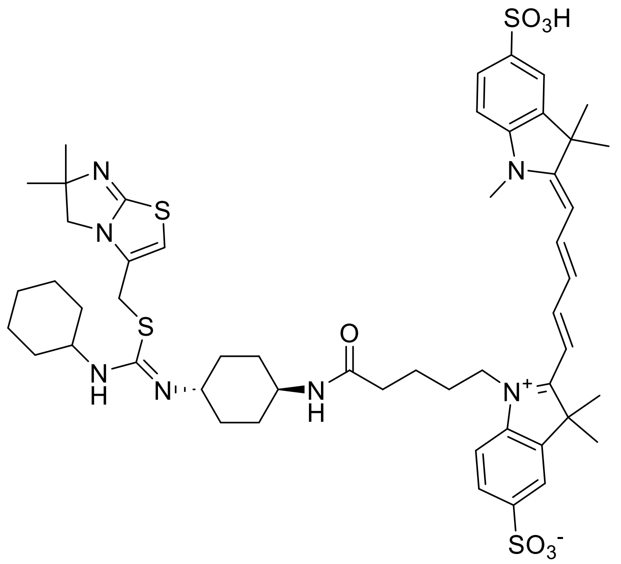

1-(6-(((1r,4r)-4-(((Z)-(cyclohexylamino)(((6,6-dimethyl-5,6-dihydroimidazo[2,1-b]thiazol-3-yl)methyl)thio)methylene)amino)cyclohexyl)amino)-6-oxohexyl)-3,3-dimethyl-2-((1E,3E)-5-((Z)-1,3,3-trimethyl-5-sulfoindolin-2-ylidene)penta-1,3-dien-1-yl)-3H-indol-1-ium-5-sulfonate

$C_{53}H_{72}N_7O_7S_4$: $[M+H]^+$ found: 1046.4

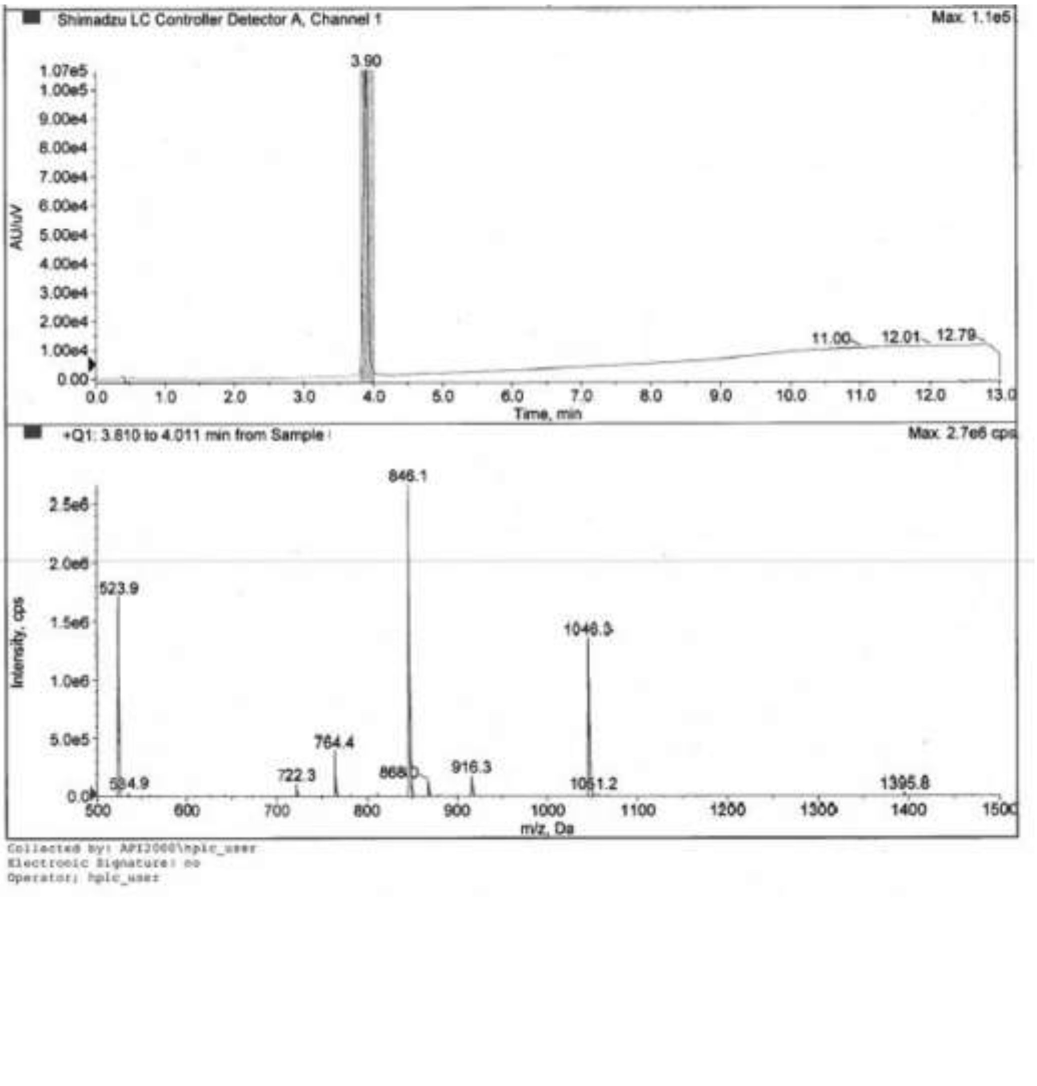

LCMS purity trace compound **18a**

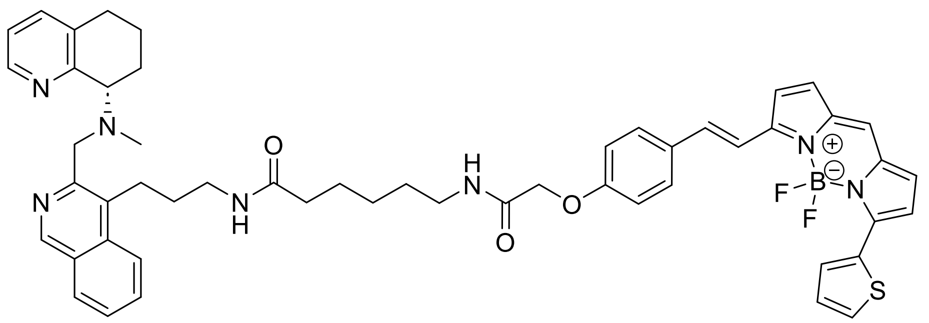

(S,E)-6-(2-(4-(2-(5,5-Difluoro-7-(thiophen-2-yl)-5H-4λ^4,5λ^4-dipyrrolo[1,2-c:2',1'-f][1,3,2]diazaborinin-3-yl)vinyl)phenoxy)acetamido)-N-(3-(3-((methyl(5,6,7,8-tetrahydroquinolin-8-yl)amino)methyl)isoquinolin-4-yl)propyl)hexanamide

$C_{52}H_{55}BF_2N_7O_3S$ $[M+H]^+$: found: 906.6

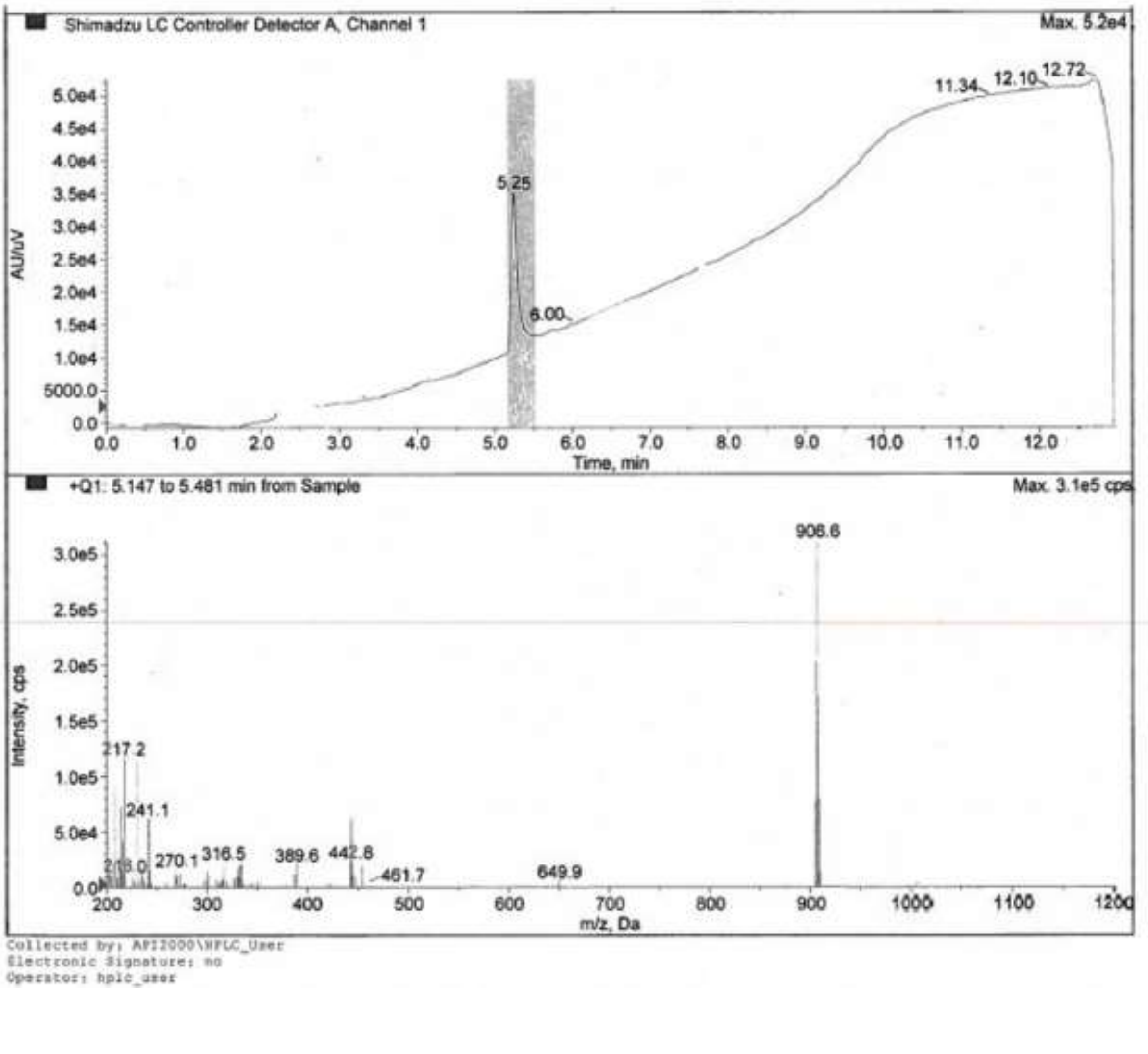

LCMS purity trace compound **18b**

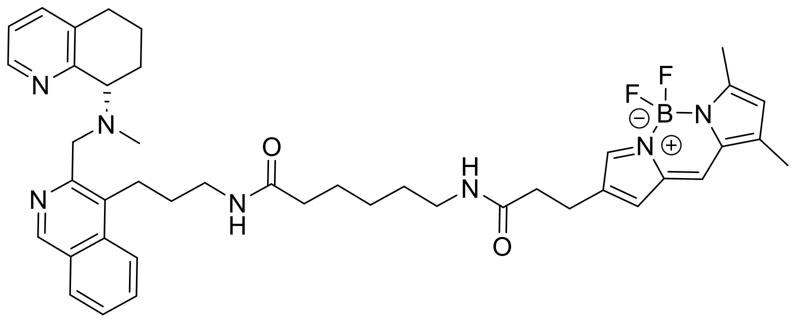

(S)-6-(3-(5,5-Difluoro-7,9-dimethyl-5H-4λ^4,5λ^4-dipyrrolo[1,2-c:2',1'-f][1,3,2]diazaborinin-2-yl)propanamido)-N-(3-(3-((methyl(5,6,7,8-tetrahydroquinolin-8-yl)amino)methyl)isoquinolin-4-yl)propyl)hexanamide

$C_{43}H_{53}BF_2N_7O_2$ $[M+H]^+$: found: 748.6

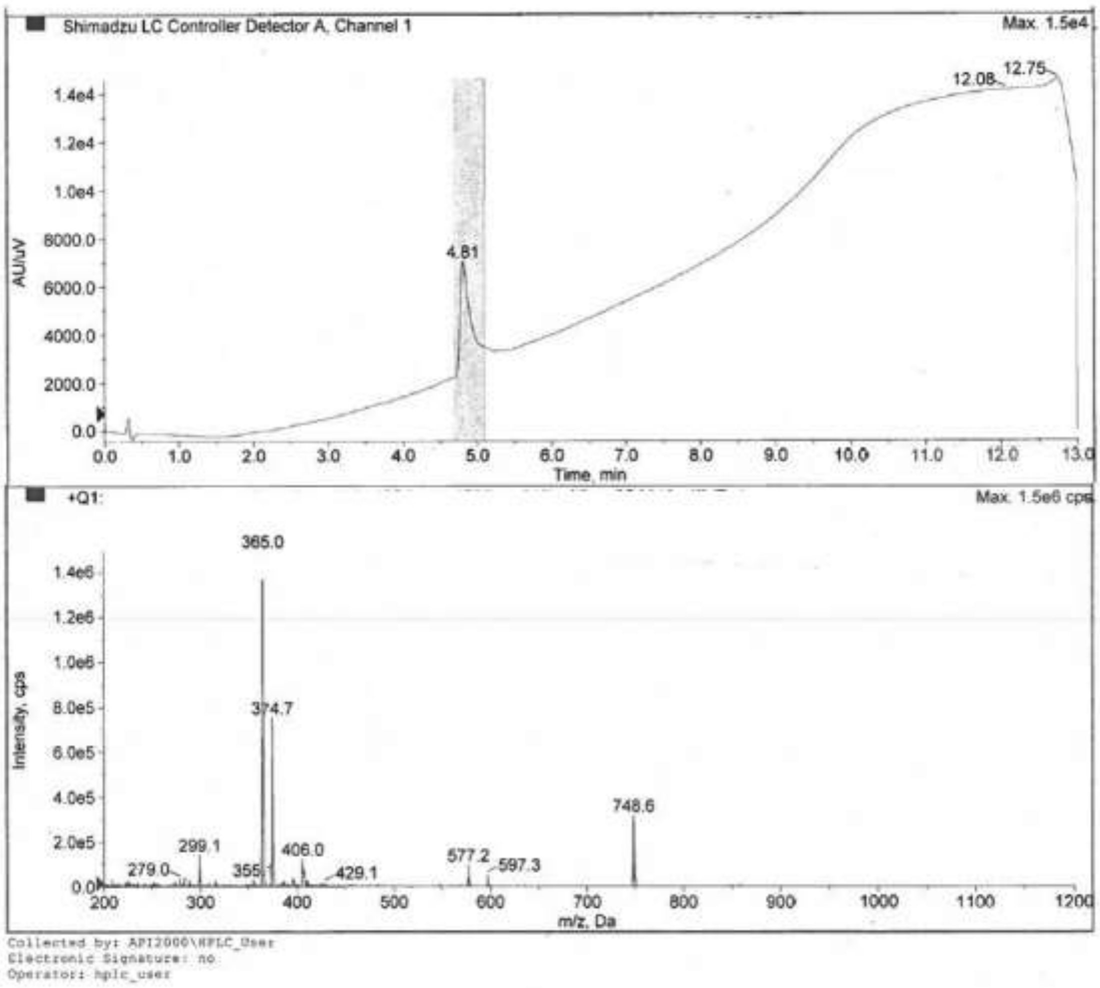

LCMS purity trace compound **24**

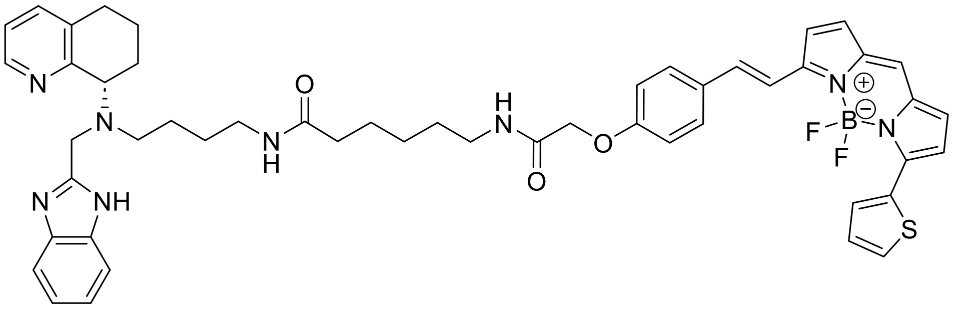

(S,E)-N-(4-(((1H-Benzo[d]imidazol-2-yl)methyl)(5,6,7,8-tetrahydroquinolin-8-yl)amino)butyl)-6-(2-(4-(2-(5,5-difluoro-7-(thiophen-2-yl)-5H-4λ4,5λ4-dipyrrolo [1,2-c:2',1'-f][1,3,2]diazaborinin-3-yl)vinyl)phenoxy)acetamido)hexanamide

$C_{50}H_{53}BF_2N_8O_3S$ $[M+H]^+$: found: 895.5

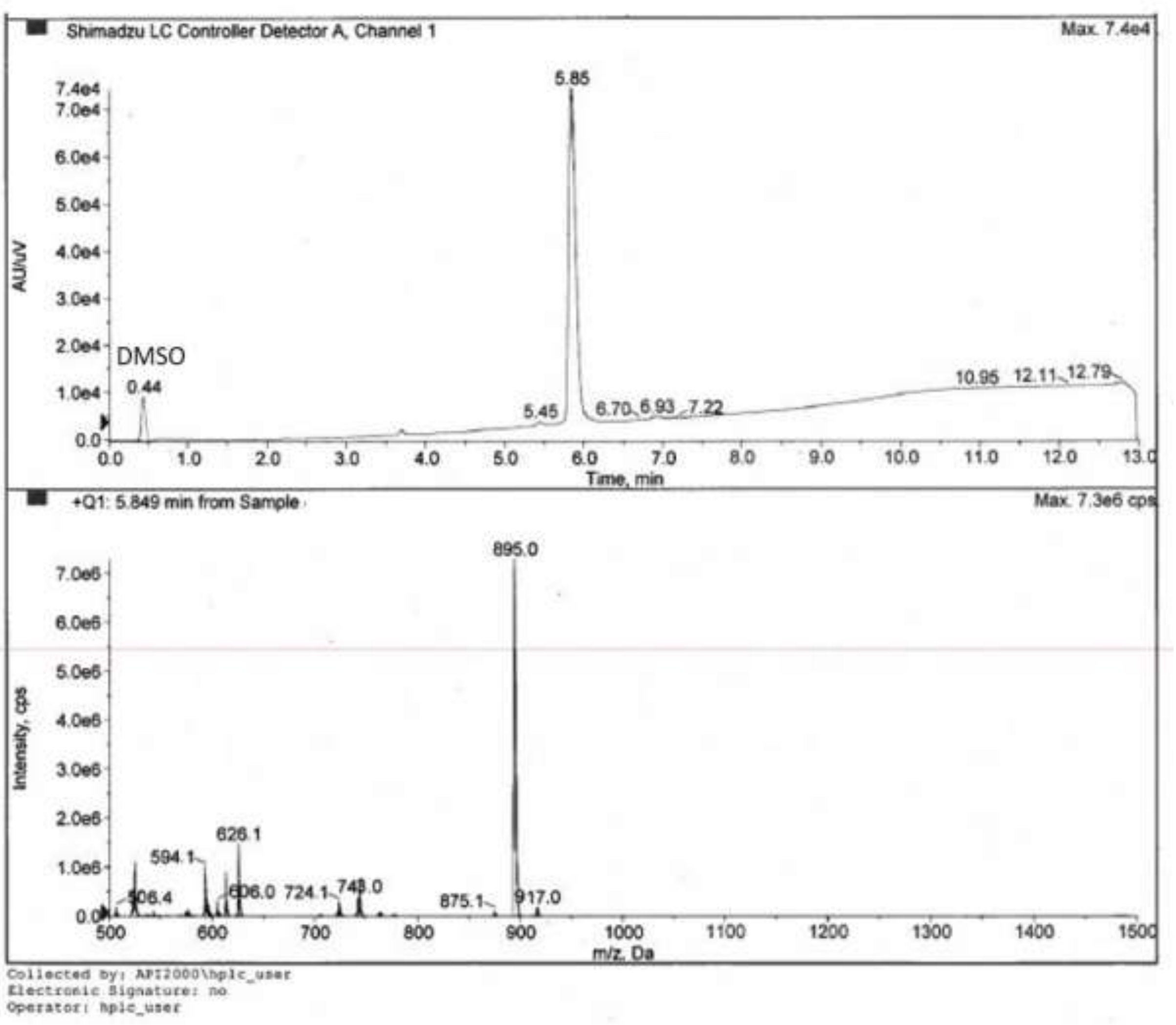

Supplement: Supplementary file 2 — jm3c00151_si_002.pdf [file jm3c00151_si_002.pdf]
